# A fine balance between Prpf19 and Exoc7 in achieving degradation of aggregated protein and suppression of cell death in spinocerebellar ataxia type 3

**DOI:** 10.1038/s41419-021-03444-x

**Published:** 2021-02-02

**Authors:** Zhefan Stephen Chen, Xiaoying Huang, Kevin Talbot, Ho Yin Edwin Chan

**Affiliations:** 1 Laboratory of Drosophila Research, School of Life Sciences, Faculty of Science, The Chinese University of Hong Kong, Shatin, N.T., Hong Kong SAR China; 2 Nexus of Rare Neurodegenerative Diseases, School of Life Sciences, Faculty of Science, The Chinese University of Hong Kong, Shatin, N.T., Hong Kong SAR China; 3 Nuffield Department of Clinical Neurosciences, University of Oxford, Oxford, UK; 4 Gerald Choa Neuroscience Centre, The Chinese University of Hong Kong, Shatin, N.T., Hong Kong SAR China

**Keywords:** Cell death in the nervous system, Spinocerebellar ataxia

## Abstract

Polyglutamine (polyQ) diseases comprise Huntington’s disease and several subtypes of spinocerebellar ataxia, including spinocerebellar ataxia type 3 (SCA3). The genomic expansion of coding *CAG* trinucleotide sequence in disease genes leads to the production and accumulation of misfolded polyQ domain-containing disease proteins, which cause cellular dysfunction and neuronal death. As one of the principal cellular protein clearance pathways, the activity of the ubiquitin–proteasome system (UPS) is tightly regulated to ensure efficient clearance of damaged and toxic proteins. Emerging evidence demonstrates that UPS plays a crucial role in the pathogenesis of polyQ diseases. Ubiquitin (Ub) E3 ligases catalyze the transfer of a Ub tag to label proteins destined for proteasomal clearance. In this study, we identified an E3 ligase, pre-mRNA processing factor 19 (Prpf19/prp19), that modulates expanded ataxin-3 (ATXN3-polyQ), disease protein of SCA3, induced neurodegeneration in both mammalian and *Drosophila* disease models. We further showed that Prpf19/prp19 promotes poly-ubiquitination and degradation of mutant ATXN3-polyQ protein. Our data further demonstrated the nuclear localization of Prpf19/prp19 is essential for eliciting its modulatory function towards toxic ATXN3-polyQ protein. Intriguingly, we found that exocyst complex component 7 (Exoc7/exo70), a Prpf19/prp19 interacting partner, modulates expanded ATXN3-polyQ protein levels and toxicity in an opposite manner to Prpf19/prp19. Our data suggest that Exoc7/exo70 exerts its ATXN3-polyQ-modifying effect through regulating the E3 ligase function of Prpf19/prp19. In summary, this study allows us to better define the mechanistic role of Exoc7/exo70-regulated Prpf19/prp19-associated protein ubiquitination pathway in SCA3 pathogenesis.

## Introduction

Polyglutamine (polyQ) diseases describe a set of neurodegenerative disorders caused by the expansion of glutamine coding *CAG* repeats in different disease genes^1^. To date, nine diseases, including Huntington’s disease, six subtypes of spinocerebellar ataxia (SCA), dentatorubral-pallidoluysian atrophy, and spinal and bulbar muscular atrophy, were reported to be caused by such triplet repeat expansion pathogenic mechanism^2^. Spinocerebellar ataxia type 3 (SCA3) is the most prevalent polyQ SCA worldwide^3^. The expansion of polyQ domain within disease protein ataxin-3 (ATXN3) causes the formation of insoluble protein aggregates in SCA3 patients^4^. The ATXN3-polyQ protein aggregates perturb a range of biological machineries, including promoting dysfunction of the ubiquitin–proteasome system (UPS) (refs. ^3,5^) and transcriptional dysregulation^6–8^. All these cellular dysfunctions hinder the survival of neurons and lead to neurodegeneration.

Several studies have demonstrated the recruitment of crucial UPS components to ATXN3-polyQ protein aggregates, suggesting a loss of UPS function in SCA3 (refs. ^9–12^). Once the function of UPS is complemented, SCA3 pathologies can be rescued^13,14^. The ubiquitin (ub) E3 ligases in the UPS confer the property of substrate recognition through mediating ubiquitination of protein substrates for proteolytic degradation^15^. Depending on the catalytic domains they exploit to exert their functions, E3 ligases can mainly be categorized into three groups, U-box, HECT, and RING-finger^16^. Several E3 ligases have been implicated in modifying SCA3 pathogenesis, targeting different species of expanded ATXN3-polyQ protein, including soluble protein^17^ and protein aggregates^18^, collectively contributing to attenuating ATXN3-polyQ protein toxicity.

Pre-mRNA-processing factor 19 (Prpf19) was first uncovered as a component in the Prpf19-Cdc5L complex, a structure involved in mediating pre-mRNA splicing inside the cell nucleus^19^. In 2001, Hatakeyama et al. demonstrated that the N-terminal U-box domain of Prpf19 possesses E3 ligase activity in vitro^20^. The U-box catalyzes the transfer of ubiquitin from ubiquitin-conjugating enzyme H3 to its substrates, and amino acid substitutions in the conserved residues within the U-box domain abolished Prpf19’s E3 function^20,21^. The structural study further reveals that the central coiled-coil domain of Prpf19 promotes homo-tetramer formation, with the dimeric U-box domains and substrate recognition WD40 domains attached to each end. This quaternary complex enables the close proximity between substrate proteins and the U-box domain to ensure efficient ubiquitination^22^. Prpf19 was shown to modulate the ubiquitination of substrates via either the nonproteolytic or the proteolytic lysine linked ubiquitin chains, the functions or expressions of ubiquitinated substrates are in turn modified^20,23–25^. Previous studies on mouse brain development suggest that Prpf19 serves as a molecular switch in governing neuron/glia differentiation, in which it inhibits neuronal differentiation while accelerating the differentiation of astrocytes^26^. Mechanistic studies further demonstrate that Prpf19 promotes the ubiquitination and degradation of protein tyrosine phosphatase 1B, a negative regulator of astrocyte differentiation^27^. These findings indicate a neuronal function of Prpf19 and highlight the involvement of its E3 function in the maturation of the mouse brain. However, the neuronal function of human Prpf19 has rarely been investigated, and its involvement in human neurological diseases remains unexplored.

In this study, we investigated the pathogenic role of Prpf19 in SCA3. We show that Prpf19 protein interacts with mutant ATXN3-polyQ protein, and targets it for ubiquitination and degradation. Using in vitro mammalian cell and in vivo *Drosophila* models, we found that overexpression of Prpf19/prp19 reduces the level of expanded ATXN3-polyQ protein, as well as ameliorating SCA3 cytotoxicities and neurodegeneration, while knockdown of *Prpf19*/*prp19* exerts an opposing effect. Most interestingly, we observed that this modulatory effect is mediated via nuclear, but not cytoplasmic Prpf19. We further demonstrated that Exoc7/exo70, an interacting partner of Prpf19/prp19, counteracts the modulatory effects of Prpf19/prp19 in terms of mediating ATXN3-polyQ protein levels and SCA3 toxicity. This study suggests the role of Prpf19 in SCA3 disease pathogenesis, and further illustrates how the regulation of Prpf19’s E3 ligase activity by Eoxc7 modifies SCA3 toxicity. Targeting this novel molecular pathway could direct the development of therapeutic interventions for SCA3.

## Materials and methods

### Plasmids

*pEGFP-ATXN3-Q28/Q78* (ref. ^18^), *pAcGFP-ATXN3-Q71* (ref. ^28^), *pcDNA3.1(+)-Q19/Q81-EGFP-myc*^29^, *EGFP-CAG27/78* (ref. ^30^), and *pCMV-flag-Htt*_*1–550*_*-Q23/Q92* (ref. ^31^) constructs were described previously. *pCMV-Prpf19*^*full-length*^*-myc/flag* was purchased from Origene (RC200810, Origene, Rockville, MD, USA). *pcDNA3.1(+)-Exoc7*^*full-length*^*-flag* was purchased from GenScript (OHu04136, GenScript, Piscataway, NJ, USA). *Exoc7*^*full-length*^*-EGFP* was a kind gift from Prof. Liwen Jiang (School of Life Sciences, The Chinese University of Hong Kong)^32^. *pcDNA3-myc-ATXN3-Q28/Q84* were gifts from Prof. Henry Paulson (Addgene plasmids # 22124 and 22125, Cambridge, MA, USA)^33^. *pRK5-HA-Ubiquitin* was a gift from Prof. Ted Dawson (Addgene plasmid # 17608)^34^. *pmCherry-N1* was a gift from Prof. Michael Davidson (Addgene plasmid # 54517). To generate the *pAcGFP-ATXN3-Q28*, the *ATXN3-Q28* DNA sequence was amplified from *pEGFP-ATXN3-Q28* plasmid using primers *KpnI-MJD-polyQ-F*, 5′-CCGGGTACCCCGCTTCGGAAGAGACGAG-3′ and *BamHI-MJD-polyQ-R*, 5′-CCGGGATCCCGGCCGCAGATCTGCTC-3′. The DNA fragment was subsequently cloned into *pAcGFP* (632470, Clontech, Mountain View, CA, USA) vector using *Kpn*I and *Bam*HI. To generate the *pcDNA3.1(+)-Prpf19*^*full-length*^*-flag*, the *Prpf19* DNA sequence was amplified from HEK293 cDNA template using primers *EcoRI-Prpf19-F*, 5′-CCGGAATTCATGTCCCTAATCTGCTCCATC-3′, and *XhoI-flag-Prpf19-R*, 5′-CCGCTCGAGTCACTTATCGTCGTCATCCTTGTAATCCATCAGGCTGTAGAACTTGAG-3′. The DNA fragment was subsequently cloned into *pcDNA3.1(+)* vector using *Eco*RI and *Xho*I. To generate the *pcDNA3.1(+)-Prpf19*^*ΔWD40*^*-flag*, *Prpf19* sequence was amplified from HEK293 cDNA template using primers *EcoRI-Prpf19-F*, 5′-CCGGAATTCATGTCCCTAATCTGCTCCATC-3′, and *XhoI-flag-Prpf19*^*1–205*^-*R*, 5′-CCGCTCGAGTCACTTATCGTCGTCATCCTTGTAATCCATGCTGAGCTCTTCTGGCTTC-3′. The DNA fragment was subsequently cloned into *pcDNA3.1(+)* vector using *Eco*RI and *Xho*I. To generate the *pcDNA3.1(+)-NES-Prpf19*^*full-length*^*-flag*, an *NES* double-stranded oligonucleotide was generated using *oligo-NES-F*, 5′-CTAGCGCCACCATGCCCGTCCCACTTCAGTTGCCTCCCTTAGAGCGTTTAACTCTAGACTGCAACGAGCCGG-3′ and *oligo-NES-R*, 5′-AATTCCGGCTCGTTGCAGTCTAGAGTTAAACGCTCTAAGGGAGGCAACTGAAGTGGGACGGGCATGGTGGCG-3′. The resultant oligonucleotide was ligated with *pcDNA3.1(+)-Prpf19*^*full-length*^*-flag*. To generate the *Prpf19*^*full-length*^*-mCherry*, *Prpf19* sequence was amplified from *pcDNA3.1(+)-Prpf19*^*full-length*^*-flag* using primers *XhoI-Prpf19-F*, 5′-CCGCTCGAGATGTCCCTAATCTGCTCCATC-3′, and *EcoRI-Prpf19-R*, 5′-CCGGAATTCGCAGGCTGTAGAACTTGAG-3′. The DNA fragment was subsequently cloned into *pmCherry-N1* vector using *Xho*I and *Eco*RI. To generate the *Prpf19*^*2X NES*^*-mCherry*, *Prpf19* sequence was amplified from *pcDNA3.1(+)-Prpf19*^*full-length*^*-flag* using primers *XhoI-Prpf19-F*, 5′-CCGCTCGAGATGTCCCTAATCTGCTCCATC-3′ and *EcoRI-2X NES-Prpf19-R*, 5′- CCGGAATTCGTAGAGTTAAACGCTCTAAGGGAGGCAACTGAAGTAGAGTTAAACGCTCTAAGGGAGGCAACTGAAGCATCAGGCTGTAGAACTT-3′. The DNA fragment was subsequently cloned into *pmCherry-N1* vector using *Xho*I and *Eco*RI. To generate the *pcDNA3.1(+)-Exoc7*^*full-length*^*-HA*, the *Exoc7* sequence was amplified from HEK293 cDNA template using primers *EcoRI-Exoc7-F*, 5′-CCGGAATTCATGATTCCCCCACAGGAGGC-3′, and *XhoI-HA-Exoc7-R*, 5′-CCGCTCGAGTCAAGCGTAATCTGGAACATCGTATGGGTAGGCAGAGGTGTCGAAAAGG-3′. The DNA fragment was subsequently cloned into *pcDNA3.1(+)* vector using *Eco*RI and *Xho*I. To generate the *pcDNA3.1(+)-Exoc7*^*ΔCC*^*-HA*, *Exoc7* sequence was amplified from HEK293 cDNA template using primers *EcoRI-Exoc7*^*1–300*^-*F*, 5′-CCGGAATTCATGATCAGAGAGGGCCCCACAGG-3′, and *XhoI-HA-Exoc7-R*, 5′-CCGCTCGAGTCAAGCGTAATCTGGAACATCGTATGGGTAGGCAGAGGTGTCGAAAAGG-3′. The DNA fragment was subsequently cloned into *pcDNA3.1(+)* vector using *Eco*RI and *Xho*I. To generate the *pcDNA3.1(+)-Exoc7*^*CC only*^*-HA*, *Exoc7* sequence was amplified from HEK293 cDNA template using primers *EcoRI-Exoc7-F*, 5′-CCGGAATTCATGATTCCCCCACAGGAGGC-3′, and *XhoI-HA-Exoc7*^*1–300*^-*R*, 5′-CCGCTCGAGTCAAGCGTAATCTGGAACATCGTATGGGTAGATCTTCTCAGTGTCACTG-3′. The DNA fragment was subsequently cloned into *pcDNA3.1(+)* vector using *Eco*RI and *Xho*I. To generate the *pcDNA3.1(+)-NES-Exoc7*^*CC only*^*-HA*, an *NES* double-stranded oligonucleotide was generated using *oligo-NES-F*, 5′-CTAGCGCCACCATGCCCGTCCCACTTCAGTTGCCTCCCTTAGAGCGTTTAACTCTAGACTGCAACGAGCCGG-3′ and *oligo-NES-R*, 5′-AATTCCGGCTCGTTGCAGTCTAGAGTTAAACGCTCTAAGGGAGGCAACTGAAGTGGGACGGGCATGGTGGCG-3′. The resultant oligonucleotide was ligated with *pcDNA3.1(+)-Exoc7*^*CC only*^*-HA*.

### Cell culture and plasmid/siRNA transfection

The human embryonic kidney 293 (HEK293) cell line (R70007, Thermo Fisher Scientific, Grand Island, NY, USA) and human neuroblastoma cell line SK-N-MC (HTB-10^TM^, American Type Culture Collection, Gaithersburg, MD, USA) were cultured using Dulbecco’s Modified Eagle’s Medium (SH30022.02, GE Healthcare Bio-Sciences, Pittsburgh, PA, USA) supplemented with 10% fetal bovine serum (F7524, Sigma-Aldrich, St. Louis, MO, USA) and 1% antibiotic–antimycotic solution (15240062, Thermo Fisher Scientific). The cells were maintained in a 37 °C humidified cell culture incubator supplemented with 5% CO_2_. Both cell lines were not recently authenticated. The mycoplasma detection was performed routinely to ensure the cells were not contaminated. Lipofectamine 2000 (11668019, Thermo Fisher Scientific) and Lipofectamine RNAiMAX (13778150, Thermo Fisher Scientific) were used in plasmid and siRNA transfection, respectively. The transfection was carried out following the manufacturer’s instructions. For the gene knockdown experiment, 5 pmol of *Prpf19*-siRNA (L-004668-00-0005, GE Healthcare Bio-Sciences) or non-targeting siRNA (D-001210-01-50, GE Healthcare Bio-Sciences) was used. Gene knockdown was performed twice prior to the plasmid transfection.

### Drug treatments

Chloroquine (C6628, Sigma-Aldrich) was used at 10 μM, and the treatment lasted 44 h. Lactacystine (SC-3575A, Santa Cruz Biotechnology, Dallas, TX, USA) was used at 5 μM, and the treatment lasted 44 h.

### Immunocytochemistry

The HEK293 or SK-N-MC cells were seeded on coverslips for 48 h (Marienfeld-Superior, Lauda-Königshofen, Germany). For the immunocytochemistry experiment shown in Fig. 6a, HEK293 cells were used and transfection lasted 24 h. For the rest of the immunocytochemistry experiments in this study, SK-N-MC cells were used and transfection lasted 48 h. Transfected cells were fixed with 3.7% paraformaldehyde for 15 min followed by permeabilization with 0.1% Triton X-100 for another 15 min. The cells were blocked with 5% goat serum at 25 °C for 1 h, followed by the incubation with primary antibody at 4 °C for 16 h. The cells were then washed three times with 1× PBS each for 5 min. The secondary antibody was used to incubate cells at 25 °C for 1 h. The cells were then washed five times with 1× PBS each for 5 min. The primary and secondary antibodies used were anti-HA (1:200; H3663, Sigma-Aldrich) and Alexa Fluor^®^ 488 AffiniPure goat anti-mouse IgG (H + L) (1:400; 115-545-062, Jackson ImmunoResearch, West Grove, PA, USA). The cell nuclei were stained with Hoechst 33342 (1:400; H-1399, Thermo Fisher Scientific) at 25 °C for 5 min. Cell images were acquired using a Zeiss LSM confocal microscope (Zeiss, Oberkochen, Germany), and images were analyzed using Fiji software (Version 2.0.0-rc-69/1.52n, NIH).

### Reverse transcription (RT)-PCR

For the preparation of fly or mammalian cell RNA samples, the total RNA was extracted from fly heads or transfected HEK293 cells using TRIzol^TM^ reagent (15596018, Thermo Fisher Scientific). The reverse transcription was performed using ImProm-II™ Reverse Transcription System (A3803, Promega, Madison, WI, USA) according to the manufacturer’s instructions. The PCR primers used were *exo70-F*, 5′-TTGGCCGAACTGAATCTTTC-3′; *exo70-R*, 5′-TGCGATCCTTATCCTTGACC-3′; *prp19-F*, 5′-GCCCGAGGATCTGGTTACAA-3′; *prp19-R*, 5′-GTATGTTCATGTCGGGCGAG-3′; *fuzzy-F*, 5′-CACATGCCATGAGTGCCTAC-3′; *fuzzy-R*, 5′-TATTAGCATGGATGCGTTGC-3′; *ATXN3-polyQ-F*, 5′-CGCGGATCCAAAAACAGCAGCAAAAGC-3′; *ATXN3-polyQ-R*, 5′-CGCACCGGTTCTGTCCTGATAGGTCC-3′; *polyQ-EGFP-F*, 5′-ATGGTCTCAACACATCACCA-3′; *polyQ-EGFP-R*: 5′-CGTCGCCGTCCAGCTCGACCAG-3′; *actin-F*, 5′-ATGTGCAAGGCCGGTTTCGC-3′; *actin-R*, 5′-CGACACGCAGCTCATTGTAG-3′.

### Immunoblotting

For the preparation of HEK293 cell protein samples, cells were transfected for 48 h followed by the lysis with SDS sample buffer (100 mM Tris-HCl, pH 6.8, 2% SDS, 40% glycerol, 5% β-mercaptoethanol, and 0.1% bromophenol blue). Samples were heated at 99 °C for 10 min prior to being subjected to the immunoblot analysis. The protein samples were then transferred to a PVDF membrane (IPVH00010, pore size 0.45 μm, Merck Millipore, Billerica, MA, USA). The membrane was blocked using 5% nonfat milk at 25 °C for 1 h, followed by the incubation of primary antibodies at 4 °C for 16 h. Primary antibodies used were anti-Prpf19 (1:1000; ab27692, Abcam, Cambridge, MA, USA), anti-cleaved caspase-3 (1:500; ab2302, Abcam), anti-myc (1:2000; 2276, Cell Signaling Technology, Danvers, MA, USA), anti-flag (1:1000; F3165, Sigma-Aldrich), anti-HA (1:1000; H3663, Sigma-Aldrich), anti-HA (1:1000; H6908, Sigma-Aldrich), anti-GFP (1:2000; 632381, Clontech) and anti-β-tubulin (1:2000; ab6046, Abcam). The membrane was washed three times with 1× TBST each for 10 min, before being subjected to the incubation of secondary antibodies at 25 °C for 1 h. Secondary antibodies used were HRP-conjugated goat anti-rabbit IgG (H + L) (1:5000, G-21234, Thermo Fisher Scientific) and HRP-conjugated goat anti-mouse IgG (H + L) (1:5000, G-21040, Thermo Fisher Scientific). The membrane was washed three times with 1× TBST each for 10 min, prior to the detection of chemiluminescence signal. The signal was developed using Immobilon Forte Western HRP substrate (WBLUF0100, Merck Millipore), and the images were captured and processed using ChemiDoc™ Touch Imaging System (170–8370, Bio-Rad, Hercules, CA, USA).

For the preparation of fly protein samples, ten fly heads from each experimental group were homogenized in 100 μl SDS sample buffer. Samples were heated at 99 °C for 10 min prior to being subjected to the immunoblot analysis. Primary antibodies used were anti-myc (1:2000; 2276, Cell Signaling Technology) and anti-β-tubulin (1:2000; ab6046, Abcam).

### Filter retardation assay

For the preparation of fly samples used for filter retardation assay, ten fly heads from each experimental group were homogenized in 100 µl filtered (16532-K, pore size 0.22 µm, Sartorius, Goettingen, Germany) 2% SDS solution, followed by the centrifugation at 16000 × *g* for 1 min to remove any debris. The samples were heated at 99 °C for 10 min, and 70 µl of each homogenate was made up to a final volume of 200 µl by 2% SDS solution before being applied to 48-well Bio-Dot^®^ Microfiltration Apparatus and filtered with cellulose acetate membrane (WHA10404131, pore size 0.2 µm, Sigma-Aldrich). Primary and secondary antibodies used were anti-myc (1:2000; 2276, Cell Signaling Technology) and HRP-conjugated goat anti-mouse IgG (H + L) (1:5000, G-21040, Thermo Fisher Scientific).

For the preparation of cell samples used for filter retardation assay, SK-N-MC cells were transfected 48 h followed by the lysis with filtered 2% SDS solution. The samples were heated at 99 °C for 10 min and diluted 20-fold with 2% SDS solution. The samples were then subjected to filter retardation assay. Primary and secondary antibodies used were anti-GFP (1:2000; 632381, Clontech) and HRP-conjugated goat anti-mouse IgG (H + L) (1:5000, G-21040, Thermo Fisher Scientific).

The chemiluminescence signal was developed using Immobilon Forte Western HRP substrate, and the images were captured and processed using ChemiDoc™ Touch Imaging System.

### Co-immunoprecipitation (co-IP) assay

The co-IP assay was performed as described previously^35^. Briefly, HEK293 cells were seeded on six-well plates, followed by gene transfection for 48 h. The transfected cells were then washed twice with ice-cold 1× PBS before the addition of binding buffer (10 mM HEPES, pH 7.5, 5 mM MgCl_2_, 142.5 mM KCl, 1 mM EDTA, 10% glycerol, and 1% Triton X-100) supplemented with protease inhibitor cocktail (P8340, Sigma-Aldrich). The sonication (duty cycle 30%, output control 3, timer 30 s, Sonifier 450, Branson Ultrasonics, Danbury, CT, USA) was performed followed by the incubation of samples at 4 °C for 1 h with gentle rotation. The samples were then centrifuged at 4 °C for 20 min at 14000 × *g*. A fraction of the supernatant was saved as “Input”. The dynabeads protein G (10004D, Thermo Fisher Scientific) was washed with a binding buffer three times. The pre-cleared beads, together with the antibodies used for immunoprecipitation were quickly applied to the remaining samples, and the mixture was incubated at 4 °C for 16 h with gentle rotation. The beads only with no primary antibody were used as a control for IP. On the following day, the beads were washed three times with binding buffer and then resuspended in SDS sample buffer. All protein samples were heated at 99 °C for 10 min, prior to immunoblotting analysis. The antibodies used for immunoprecipitation (IP) and immunoblotting (IB) are summarized in Supplementary Table 1.

### In vitro ubiquitination assay

The HEK293 cells were treated with 5 μM of MG-132 (474790, Merck Millipore) before harvested using 100 μl ubiquitination lysis buffer (10 mM Tris-HCl, pH 8.0, 150 mM NaCl, 2% SDS) with protease inhibitor cocktail (P8340, Sigma-Aldrich) freshly added. The homogenate was heated at 99 °C for 10 min followed by sonication (duty cycle 20%, output control 2, timer 10 s, signifier 450, Branson Ultrasonic). Another 900 μl of ubiquitination dilution buffer (10 mM Tris-HCl, pH 8.0, 150 mM NaCl, 2 mM EDTA, and 1% Triton X-100) was added to each sample followed by rotation at 4 °C for 1 h. A volume of 50 μl of homogenate was saved as “input”. The dynabeads protein G (10004D, Thermo Fisher Scientific) was washed with a binding buffer three times. The pre-cleared beads, together with the antibodies used for immunoprecipitation were quickly added to the remaining samples, and the mixture was incubated at 4 °C for 16 h with gentle rotation. The beads only with no primary antibody were used as a control for IP. The beads were washed three times with ubiquitination washing buffer (10 mM Tris-HCl, pH 8.0, 1 M NaCl, 1 mM EDTA, and 1% NP-40) and then resuspended in SDS sample buffer. All samples were heated at 99 °C for 10 min, prior to immunoblot analysis. The antibodies used for immunoprecipitation (IP) and immunoblotting (IB) are summarized in Supplementary Table 2.

### *Drosophila* genetics

All fly stocks and genetic crosses were maintained in 21.5 °C incubators. Fly lines *gmr-Gal4* (ref. ^36^), *elav-Gal4* (ref. ^37^), *UAS-ATXN3fl-Q27/Q84* (ref. ^38^), and *UAS-ATXN3tr-Q27/Q78* (ref. ^39^) were described previously. The *UAS-prp19-dsRNA*^*GD22147*^, *UAS-prp19-dsRNA*^*GD41438*^, and *UAS-exo70-dsRNA*^*GD12140*^ lines were obtained from Vienna *Drosophila* RNAi Center, and the *prp19*^*G3080*^ (28455) line was obtained from Bloomington *Drosophila* Stock Center. Both male and female flies were used in all experiments. No randomization and blinding were done.

### Pseudo pupil analysis

The pseudo pupil analysis was performed as described previously^40^. Briefly, fly heads were decapitated and mounted on a glass slide with a drop of immersion oil. The eyes were examined under a light microscope (CX31; Olympus) using a ×60 oil objective. The images of ommatidia were captured by a SPOT Insight CCD camera (Diagnostic Instruments Inc.), and all images were processed using the SPOT Advanced software (Version 5.2; Diagnostic Instruments Inc.) and Adobe Photoshop 7.0 software. A total of 200 ommatidia were examined for each control or experimental group. The average number of rhabdomeres per ommatidia was used to determine ommatidial integrity.

### Climbing and survival analyses

The climbing and survival analyses were performed as described previously^41,42^. For climbing analysis, flies were anesthetized and put into a vertical plastic column. After 1 h of the recovery period, flies were banged to the bottom, and the climbing activity was measured by calculating the percentage of flies that failed to climb (<6 cm) in 10 s. Six trials were performed at 3 min of intervals in each control or experimental group.

### Statistical analyses

The two-tailed, unpaired Student’s *t* test was used for the comparison between two experimental groups. One-way ANOVA followed by post hoc Tukey’s test was applied to determine the difference between each experimental group when comparing three or more groups. Log-rank (Mantel–Cox) test was used for survival analysis. *, **, ***, and **** represent *P* < 0.05, *P* < 0.01, *P* < 0.001, and *P* < 0.0001, respectively, which are considered statistically significant. ns indicates no significant difference. GraphPad Prism version 9.0.0 was used for statistical analysis.

## Results

### Prpf19 promotes the ubiquitination and degradation of expanded ATXN3-polyQ protein and suppresses cytotoxicity in ATXN3-polyQ-expressing cells

**Fig. 1:**
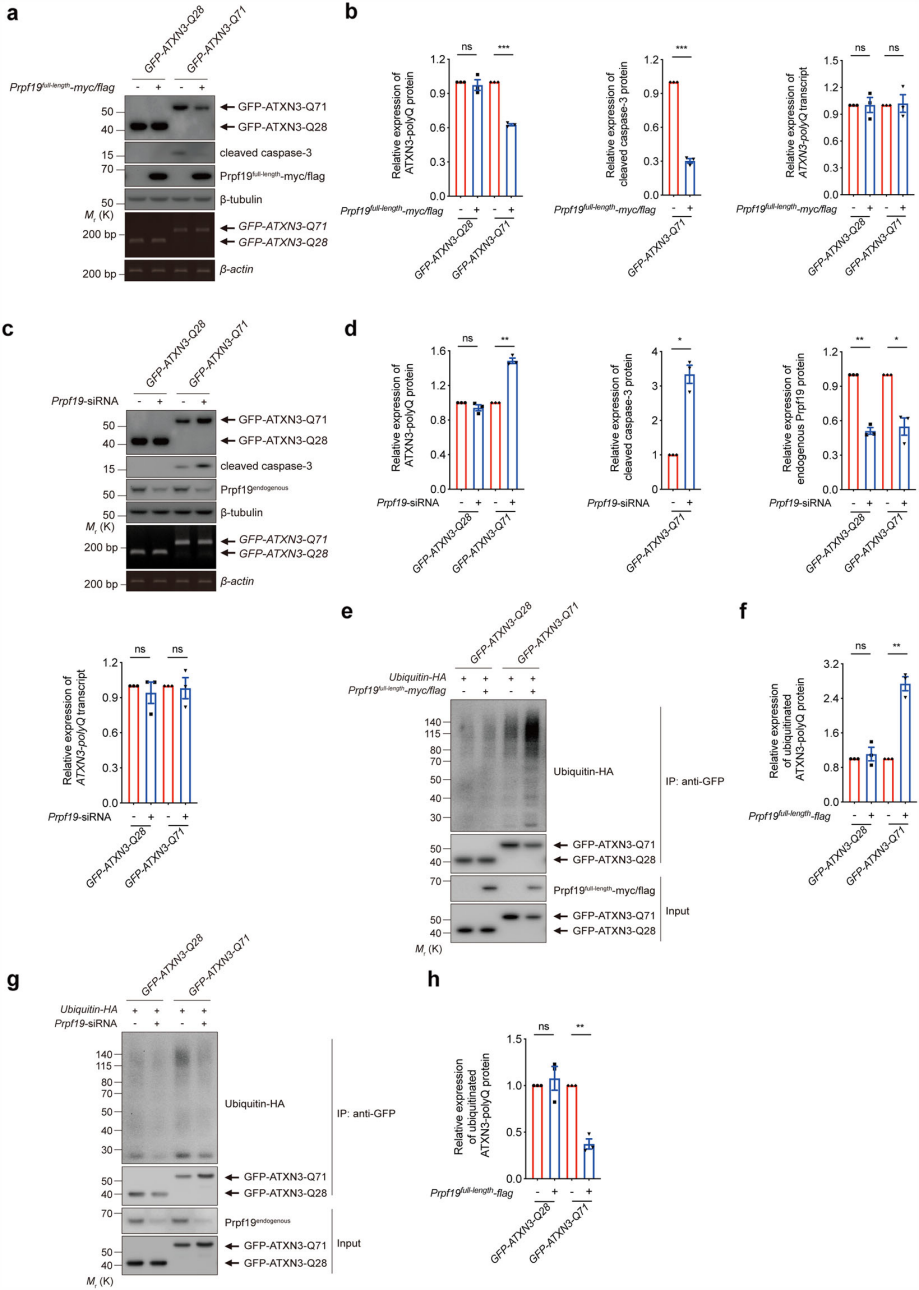
Prpf19 modulates the poly-ubiquitination and degradation of expanded ATXN3-polyQ protein and also mediates ATXN3-polyQ-induced cytotoxicity. **a** Overexpression of Prpf19 degraded the level of ATXN3-Q71 and suppressed the ATXN3-Q71-induced caspase-3 cleavage. The expression of ATXN3-Q28 was not affected, and the transcript level of *ATXN3-polyQ* was not altered when Prpf19 was overexpressed. *n* = 3 biological replicas. Each *n* represents an independent preparation of cell protein and RNA samples. **b** Quantification of ATXN3-polyQ protein, cleaved caspase-3 protein, and *ATXN3-polyQ* transcript levels in panel **a**. Error bars represent S.E.M. Statistical analysis was performed using two-tailed unpaired Student’s *t* test. ns denotes no significant difference and *** denotes *P* < 0.001. **c** Knockdown of *Prpf19* increased the level of ATXN3-Q71 and enhanced the ATXN3-Q71-induced caspase-3 cleavage. The expression of ATXN3-Q28 was not affected, and the transcript level of *ATXN3-polyQ* was not altered when *Prpf19* was knocked down. *n* = 3 biological replicas. Each *n* represents an independent preparation of cell protein and RNA samples. **d** Quantification of ATXN3-polyQ protein, cleaved caspase-3 protein, endogenous Prpf19 protein, and *ATXN3-polyQ* transcript levels in panel **c**. Error bars represent S.E.M. Statistical analysis was performed using two-tailed unpaired Student’s *t* test. ns denotes no significant difference, * denotes *P* < 0.05 and ** denotes *P* < 0.01. **e** Overexpression of Prpf19 promoted the poly-ubiquitination of ATXN3-Q71, and the level of ubiquitinated ATXN3-Q28 was not changed. *n* = 3 biological replicas. Each *n* represents an independent preparation of cell protein samples. **f** Quantification of the ubiquitination level of ATXN3-polyQ protein in panel **e**. Error bars represent S.E.M. Statistical analysis was performed using two-tailed unpaired Student’s *t* test. ns denotes no significan*t* difference and ** denotes *P* < 0.01. **g** Knockdown of *Prpf19* reduced the poly-ubiquitination of ATXN3-Q71 protein, and the level of ubiquitinated ATXN3-Q28 was not changed. *n* = 3 biological replicas. Each *n* represents an independent preparation of cell protein samples. **h** Quantification of the ubiquitination level of ATXN3-polyQ protein in panel **g**. Error bars represent S.E.M. Statistical analysis was performed using two-tailed unpaired Student’s *t* test. ns denotes no significant difference and ** denotes *P* < 0.01. Beta*-*actin or beta-tubulin was used as a loading control. Only representative gels and blots are shown.

Since Prpf19 protein was previously reported to interact with expanded *huntingtin exon1 CAG* transcript^43^, we tested if Prpf19 plays any role in mediating RNA toxicity in polyQ disease models. Using a cell-based model^30^, we showed that overexpression of Prpf19 did not modulate the caspase-3 activation elicited from the expression of elongated *polyQ* RNA (Supplementary Fig. 1). Interestingly, by means of co-immunoprecipitation, we found that polyQ protein that carries an elongated polyQ domain physically interacts with endogenous Prpf19 protein in HEK293 cells (Supplementary Fig. 3a). Next, we overexpressed Prpf19 in ATXN3-polyQ-expressing cells to investigate whether Prpf19 exerts any modulatory effect on polyQ protein toxicity. It was found that Prpf19 overexpression reduced the level of ATXN3-Q71 (Fig. 1a, b). Caspase-3 activation was detected in ATXN3-Q71-expressing cells as indicated by caspase-3 cleavage (Fig. 1a, b). We observed that caspase-3 activation was suppressed upon Prpf19 overexpression (Fig. 1a, b). In contrast, knockdown of *Prpf19* expression led to an increased cellular level of ATXN3-Q71 protein and enhancement of caspase-3 cleavage (Fig. 1c, d). As a control, the expression of unexpanded polyQ protein ATXN3-Q28 was not altered upon either overexpression or knockdown of *Prpf19* (Fig. 1a–d). Meanwhile, the modulatory effect of Prpf19 was further tested on Htt_1–550_-polyQ (Supplementary Fig. 2a, b) and polyQ-EGFP (Supplementary Fig. 3b–e) models. Similar effects were detected. Taken together, our findings demonstrate that Prpf19 physically interacts with expanded polyQ protein and modifies expanded polyQ protein-induced cytotoxicity.

Given that Prpf19 protein possesses ubiquitin (ub) E3 ligase activity, we argued that if polyQ protein ubiquitination would be modulated by Prpf19. We immunoprecipitated ATXN3-polyQ proteins and determine their levels of ubiquitination. In the unexpanded ATXN3-Q28 control, only a low level of ubiquitinated polyQ protein was detected and overexpression or knockdown of *Prpf19* did not alter ATXN3-Q28 ubiquitination level (Fig. 1e–h). In contrast, more intense ubiquitination was observed in the expanded ATXN3-Q71 protein. Upon overexpression and knockdown of *Prpf19*, the ubiquitination level of ATXN3-Q71 was increased and decreased, respectively (Fig. 1e–h). The modulatory effect of Prpf19 on expanded polyQ protein ubiquitination was further validated on Htt_1–550_-polyQ (Supplementary Fig. 2c, d) and polyQ-EGFP (Supplementary Fig. 3f, g) models.

### The WD40 domain of Prpf19 protein is responsible for exerting its modulatory effect on mutant ATXN3-polyQ protein

**Fig. 2:**
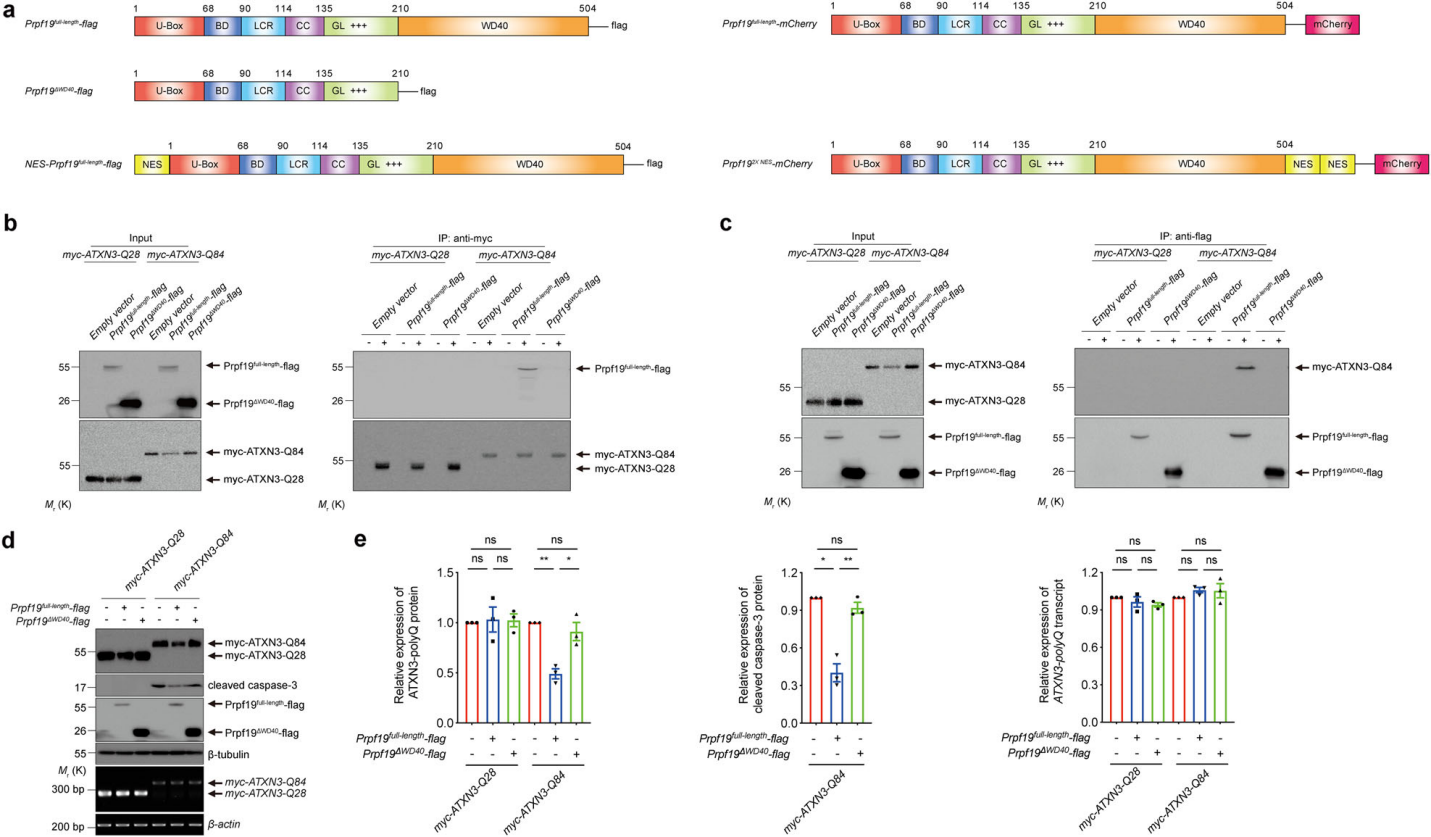
The WD40 domain of Prpf19 protein is responsible for the degradation of expanded ATXN3-polyQ protein, and suppression of ATXN3-polyQ-induced cytotoxicity. **a** Schematic representation of the domain composition of Prpf19 protein. U-box U-box domain, BD binding domain, LCR low complexity region, CC coiled-coil domain, GL globular domain, +++ charged region, WD40 WD40 domain. A nuclear export signal (NES) protein sequence was attached to the N-terminus of Prpf19^full-length^ to generate NES-Prpf19^full-length^. A Flag tag was added to the C-terminus of Prpf19^full-length^, Prpf19^ΔWD40^, and NES-Prpf19^full-length^, respectively. Prpf19^full-length^ protein sequence was inserted before the mCherry to generate Prpf19^full-length^-mCherry. Two NES protein sequences were inserted between Prpf19^full-length^ and mCherry sequences to generate Prpf19^2X NES^-mCherry. **b** Removal of the WD40 domain disrupted the interaction between Prpf19 and ATXN3-Q84 protein. “+” indicates that the anti-myc antibody was present in the immunoprecipitant, while “−” indicates that the anti-myc antibody was not included in the immunoprecipitant. *n* = 3 biological replicas. Each *n* represents an independent preparation of cell protein samples. **c** The interaction between Prpf19 and expanded ATXN3-Q84 protein was abolished when the WD40 domain was deleted. “+” indicates that the anti-flag antibody was present in the immunoprecipitant, while “−” indicates that the anti-flag antibody was not included in the immunoprecipitant. *n* = 3 biological replicas. Each *n* represents an independent preparation of cell protein samples. **d** Overexpression of Prpf19^full-length^, but not Prpf19^ΔWD40^, reduced the level of expanded ATXN3-Q84 protein and suppressed the ATXN3-Q84-induced caspase-3 cleavage. Overexpression of neither Prpf19^full-length^ nor Prpf19^ΔWD40^ alters the level of *ATXN3-Q28* or *ATXN3-Q84* transcript. *n* = 3 biological replicas. Each *n* represents an independent preparation of cell protein and RNA samples. **e** Quantification of ATXN3-polyQ protein, cleaved caspase-3 protein, and *ATXN3-polyQ* transcript levels in panel **d**. Error bars represent S.E.M. Statistical analysis was performed using one-way ANOVA followed by post hoc Tukey’s test. ns denotes no significant difference, * denotes *P* < 0.05, and ** denotes *P* < 0.01. Beta-actin or beta-tubulin was used as a loading control. Only representative gels and blots are shown.

The Prpf19 protein is a multi-domain protein, which contains a U-box domain, a binding domain, a low complexity region, a coiled-coil domain, a globular domain, a charged region, and a WD40 domain (ref. ^44^; Fig. 2a). As demonstrated in co-immunoprecipitation analysis, the WD40 domain is responsible for mediating the interaction between Prpf19 and mutant ataxin-3 protein, ATXN3-Q84 (Fig. 2b, c). However, no such interaction was observed between Prpf19 or its WD40 deletion mutant (Prpf19^ΔWD40^) and unexpanded ATXN3-Q28 protein (Fig. 2b, c), suggesting that the WD40 domain in Prpf19 specifically interacts with mutant ATXN3-polyQ protein. We next decided to examine whether the WD40 domain in Prpf19 is essential for modulating the level of ATXN3-polyQ protein in cells. We found that overexpression of Prpf19^ΔWD40^ deletion protein failed to reduce ATXN3-Q84 protein expression and suppress caspase-3 cleavage induced by the toxic ATXN3-Q84 disease protein (Fig. 2d, e). The above findings clearly demonstrate that Prpf19 interacts with and targets mutant ATXN3-polyQ protein for degradation via its WD40 domain.

### The function of the proteasome is required for Prpf19 to exert its modifying effect on ATXN3-polyQ disease protein

**Fig. 3:**
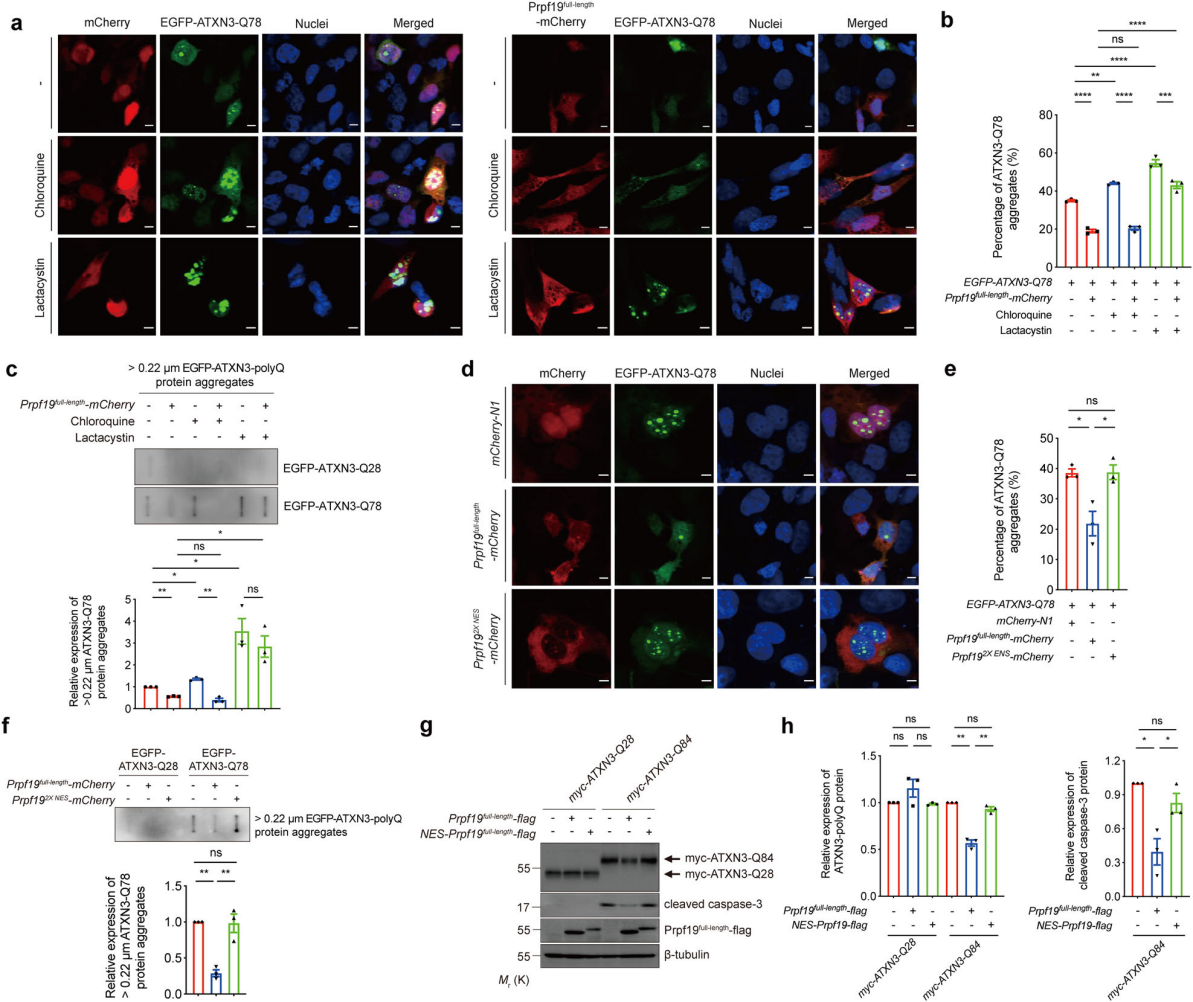
Inhibition of proteasomal function partially disrupts the effect of Prpf19 on degrading ATXN3-polyQ protein aggregates, and the nuclear Prpf19 is essential for mediating the degradation of expanded ATXN3-polyQ protein, and suppression of ATXN3-polyQ-induced cytotoxicity. **a** Treatment of chloroquine or lactacystin increased the percentage of cells with ATXN3-Q78 (green) protein aggregates. Overexpression of Prpf19 (red) reduced the percentage of cells with ATXN3-Q78 (green) protein aggregates either in the absence or presence of chloroquine. However, treatment of lactacystin abolished the effect of Prpf19 on modulating ATXN3-Q78 protein aggregation. Cell nuclei (blue) were stained with Hoechst 33342. Scale bars: 5 μm. *n* = 3 biological replicas. Each *n* represents an independent preparation of immunocytochemistry samples. **b** Quantification of the percentage of cells with ATXN3-Q78 aggregates in panel **a**. At least 100 cells were counted in each control or experimental group in an independent experiment. Error bars represent S.E.M. Statistical analysis was performed using one-way ANOVA followed by post hoc Tukey’s test. ns denotes no significant difference, ** denotes *P* < 0.01, *** denotes *P* < 0.001, and **** denotes *P* < 0.0001. **c** Overexpression of Prpf19^full-length^ reduced the level of > 0.22 µm ATXN3-Q78 protein aggregates, while such modulatory effect was abolished when cells were treated with lactacystin, but not chloroquine. *n* = 3 biological replicas. Each *n* represents an independent preparation of cell protein samples. Error bars represent S.E.M. Statistical analysis was performed using one-way ANOVA followed by post hoc Tukey’s test. ns denotes no significant difference, * denotes *P* < 0.05, and ** denotes *P* < 0.01. **d** The Prpf19^full-length^ (red) protein localized in both nuclear and cytoplasmic compartments, while the Prpf19^2X NES^ (red) protein predominantly localized in the cytoplasm. Overexpression of Prpf19^full-length^, but not Prpf19^2X NES^ reduced the percentage of cells with ATXN3-Q78 (green) protein aggregates. Cell nuclei (blue) were stained with Hoechst 33342. Scale bars: 5 μm. *n* = 3 biological replicas. Each *n* represents an independent preparation of immunocytochemistry samples. **e** Quantification of the percentage of cells with ATXN3-Q78 aggregates in panel **c**. At least 100 cells were counted in each control or experimental group in an independent experiment. Error bars represent S.E.M. Statistical analysis was performed using one-way ANOVA followed by post hoc Tukey’s test. ns denotes no significant difference and * denotes *P* < 0.05. **f** Overexpression of Prpf19^full-length^, but not Prpf19^2X NES^ reduced the level of > 0.22 µm ATXN3-Q78 protein aggregates. *n* = 3 biological replicas. Each *n* represents an independent preparation of cell protein samples. Error bars represent S.E.M. Statistical analysis was performed using one-way ANOVA followed by post hoc Tukey’s test. ns denotes no significant difference and ** denotes *P* < 0.01. **g** Overexpression of Prpf19, but not NES-Prpf19, led to the degradation of ATXN3-Q84 protein, and suppression of ATXN3-Q84-induced caspase-3 cleavage. Overexpression of neither Prpf19 nor NES-Prpf19 caused the change in the level of ATXN3-Q28 protein. *n* = 3 biological replicas. Each *n* represents an independent preparation of cell protein samples. **h** Quantification of the ATXN3-polyQ protein and cleaved caspase-3 protein levels in panel **g**. Error bars represent S.E.M. Statistical analysis was performed using one-way ANOVA followed by post hoc Tukey’s test. ns denotes no significant difference, * denotes *P* < 0.05 and ** denotes *P* < 0.01. Beta-tubulin was used as a loading control. Only representative images and blots are shown.

When expressed in neuronal cell line SK-N-MC, mutant ATXN3-polyQ protein forms microscopic protein aggregates (ref. ^45^; Fig. 3a). We next sought to determine whether Prpf19 exerts a modulatory effect on ATXN3-polyQ protein aggregation. We found that overexpression of Prpf19 reduced the percentage of cells with EGFP-ATXN3-Q78 protein aggregates (Fig. 3a, b). To further investigate whether the lysosomal and UPS protein degradation pathways take part in the process of Prpf19-mediated reduction on polyQ protein aggregation, we treated Prpf19 and EGFP-ATXN3-Q78 co-expressing cells with the lysosomal inhibitor chloroquine or the proteasome inhibitor lactacystin. Similar to what was reported previously^18^, treatment of either chloroquine or lactacystin increased the number of polyQ protein aggregates in cells (ref. ^18^; Fig. 3a, b). In our study, overexpression of Prpf19 was still able to reduce protein aggregation in the presence of chloroquine, while the Prpf19’s modifying effect was diminished in lactacystin-treated cells (Fig. 3a, b). In addition to fluorescence microscopic analysis, we further analyzed polyQ protein aggregation using a semi-quantitative filter retardation assay. Overexpression of Prpf19 reduced the amount of EGFP-ATXN3-Q78 protein that is large than 0.22 µm, while such effect was abolished upon treatment of lactacystin but not chloroquine (Fig. 3c). These results indicate that normal proteasomal function is required for Prpf19 to exert its modulatory effect on ATXN3-polyQ protein aggregation.

### Nuclear Prpf19 elicits modulatory effect on ATXN3-polyQ disease protein

When overexpressed in cells, Prpf19 was found localized in both the nuclear and cytoplasmic compartments (Fig. 3d and Supplementary Fig. 4). We, therefore, sought to investigate which subcellular compartment is critical for Prpf19 to elicit its modulatory effect on ATXN3-polyQ protein aggregation and toxicity. We generated a cytosolic version of Prpf19 (Prpf19^2X NES^) by introducing exogenous nuclear export signal (NES) sequences to the protein (Fig. 2a). The addition of NES sequences caused the Prpf19^2XNES^ protein to localize predominantly to the cytoplasm (Fig. 3d and Supplementary Fig. 4). Interestingly, we found that cytoplasmic Prpf19 was incapable of executing its modulatory effect on ATXN3-polyQ protein aggregation (Fig. 3d–f). Consistently, cytoplasmic Prpf19 also failed to modify SDS-soluble ATXN3-Q84 protein level and suppress caspase-3 activation (Fig. 3g, h). This indicates that nuclear localization is essential for Prpf19 to target ATXN3-polyQ protein aggregation and toxicity.

### Exoc7 counteracts the effect of Prpf19 on ATXN3-polyQ protein degradation and suppression of ATXN3-polyQ toxicity

**Fig. 4:**
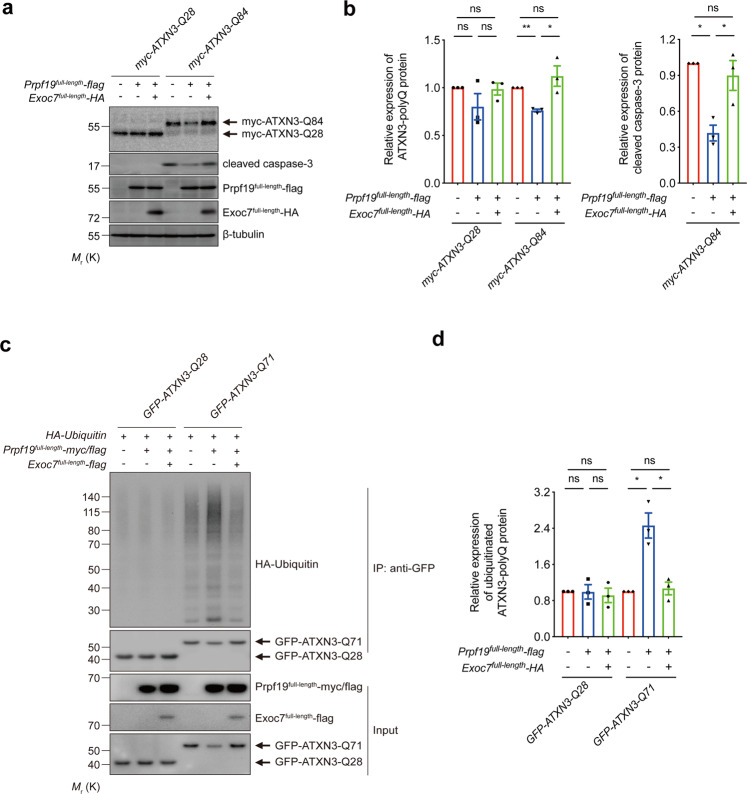
Overexpression of Exoc7 perturbs the Prpf19-mediated expanded ATXN3-polyQ protein ubiquitination, degradation, and suppression of ATXN3-polyQ-induced cytotoxicity. **a** Reduction of ATXN3-Q84 protein level and cytotoxicity mediated by Prpf19 overexpression was resumed when Exoc7 was co-overexpressed. *n* = 3 biological replicas. Each *n* represents an independent preparation of cell protein samples. **b** Quantification of ATXN3-polyQ protein and cleaved caspase-3 protein levels in panel **a**. Error bars represent S.E.M. Statistical analysis was performed using one-way ANOVA followed by post hoc Tukey’s test. ns denotes no significant difference, * denotes *P* < 0.05, and ** denotes *P* < 0.01. **c** Overexpression of Prpf19 promoted the poly-ubiquitination of ATXN3-Q71 protein, whereas the ubiquitination level of ATXN3-Q28 protein was not altered. Co-overexpression of Exoc7 suppressed the Prpf19-mediated increased poly-ubiquitination of ATXN3-Q71 protein. Meanwhile, the ubiquitination level of ATXN3-Q28 protein was not affected. *n* = 3 biological replicas. Each *n* represents an indepe*n*dent preparation of cell protein samples. **d** Quantification of the ubiquitinated ATXN3-polyQ protein levels in panel **c**. Error bars represent S.E.M. Statistical analysis was performed using one-way ANOVA followed by post hoc Tukey’s test. ns denotes no significant difference and * denotes *P* < 0.05. Beta-tubulin was used as a loading control. Only representative blots are shown.

It has been reported that Prpf19 physically interacts with exocyst complex component 7 (Exoc7), a protein involved in exocytosis^46^, and such interaction interferes with Prpf19-mediated pre-mRNA splicing^44^. This demonstrates that Exoc7 plays a negative regulatory role in Prpf19 functioning. We investigated whether the co-expression of Exoc7 would modify Prpf19’s modulatory effect on ATXN3-polyQ protein toxicity. We found that the co-expression of full-length Exoc7 counteracted Prpf19’s ability in reducing ATXN3-Q84 protein level, as well as suppressing ATXN3-Q84-induced caspase-3 cleavage (Fig. 4a, b). Prpf19 overexpression promoted poly-ubiquitination of expanded ATXN3-polyQ protein (Fig. 1d), and such effect was abolished upon Exoc7 co-expression (Fig. 4c, d). As a control, the level of unexpanded ATXN3-polyQ protein remained unaltered regardless of Prpf19 and Exoc7 co-expression (Fig. 4a–d). These findings suggest that Exoc7 serves as a negative regulator of Prpf19, and the effect is specific for mutant ATXN3-polyQ protein.

### The nuclear-localized Exoc7^CC only^ inhibits the E3 activity of Prpf19

**Fig. 5:**
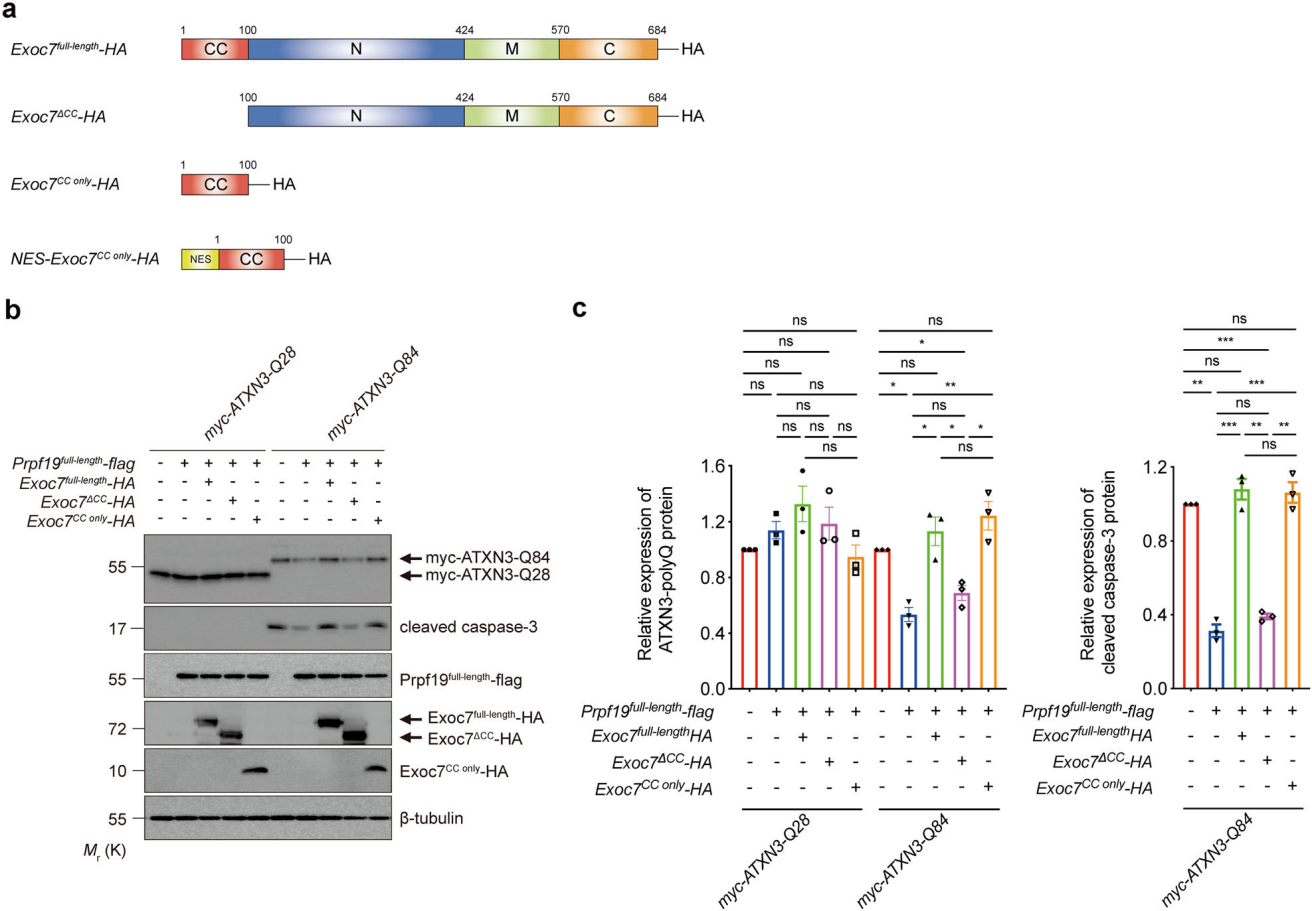
Overexpression of Exoc7 coiled-coil domain perturbs the Prpf19-mediated expanded ATXN3-polyQ protein degradation and suppression of ATXN3-polyQ-induced cytotoxicity. **a** Schematic representation of the domain composition of Exoc7 protein. CC coiled-coil domain, N N-terminal domain, M middle domain, C C-terminal domain. A nuclear export signal (NES) protein sequence was attached to the N-terminus of Exoc7^CC only^ to generate NES-Exoc7^CC only^. A HA tag was added to the C-terminus of Exoc7^full-length^, Exoc7^ΔCC^, Exoc7^CC only^, and NES-Exoc7^CC only^, respectively. **b** Overexpression of Prpf19 reduced the expression of ATXN3-Q84 protein and suppressed ATXN3-Q84-induced caspase-3 cleavage. Co-overexpression of either Exoc7^full-length^ or Exoc7^CC only^, but not Exoc7^ΔCC^, abolished the Prpf19-mediated ATXN3-Q84 protein degradation and suppression of ATXN3-Q84-induced cytotoxicity. *n* = 3 biological replicas. Each *n* represents an independent preparation of cell protein samples. **c** Quantification of ATXN3-polyQ protein and cleaved caspase-3 protein levels in panel **b**. Error bars represent S.E.M. Statistical analysis was performed using one-way ANOVA followed by post hoc Tukey’s test. ns denotes no significant difference, * denotes *P* < 0.05, ** denotes *P* < 0.01, and *** denotes *P* < 0.001. Beta-tubulin was used as a loading control. Only representative blots are shown.

**Fig. 6:**
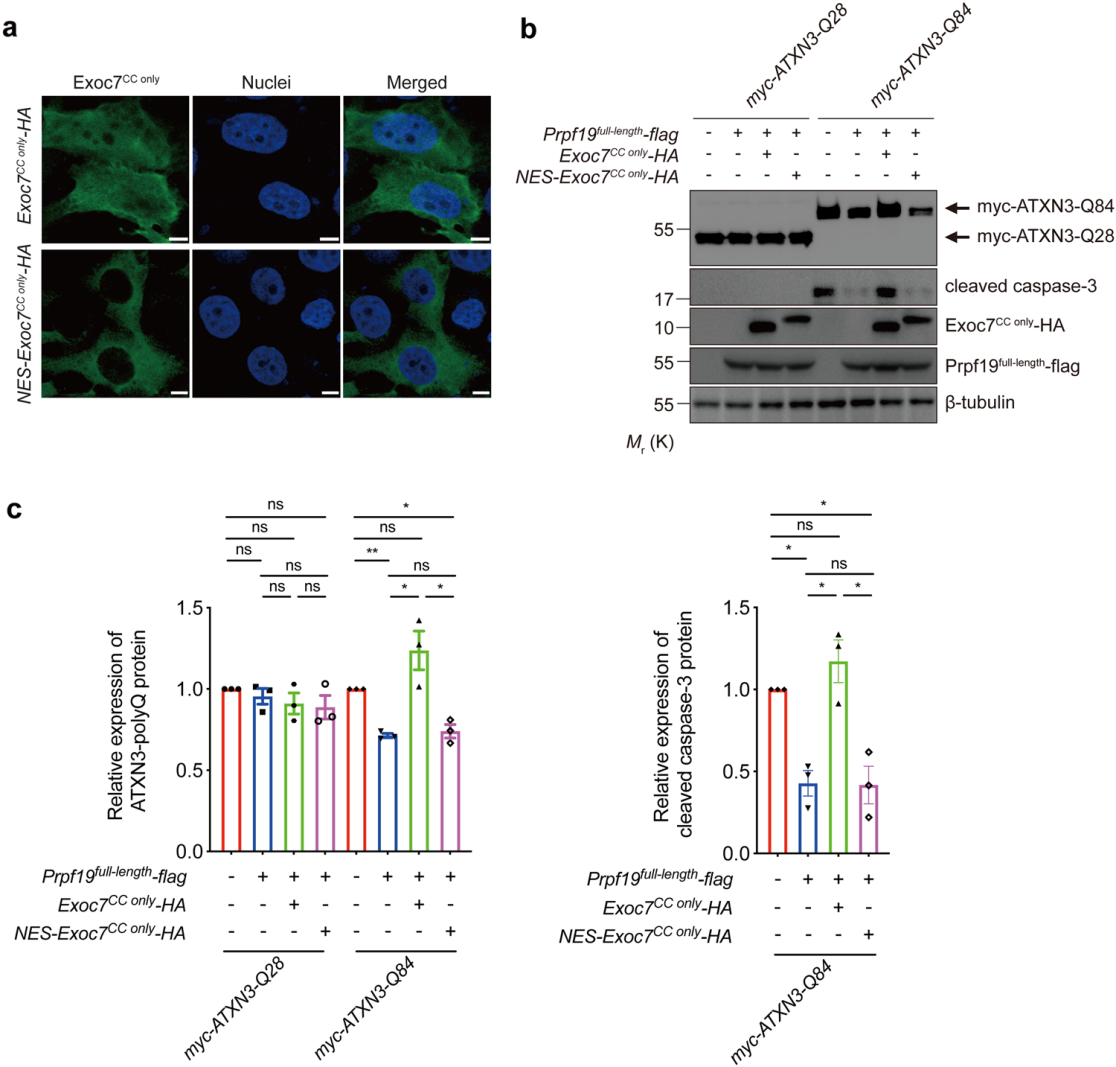
The nuclear Exoc7^CC only^ is responsible for counteracting the Prpf19-mediated expanded ATXN3-polyQ degradation and suppression of ATXN3-polyQ-induced cytotoxicity. **a** The Exoc7^CC only^ (green) localized evenly in both the nuclear and cytoplasmic compartments, while the addition of the NES protein sequence caused the cytoplasmic retention of Exoc7^CC only^. Cell nuclei (blue) were stained with Hoechst 33342. Scale bars: 5 μm. *n* = 3 biological replicas. Each *n* represents an independent preparation of immunocytochemistry samples. **b** Overexpression of Prpf19 led to the reduction of ATXN3-Q84 protein level and suppression of ATXN3-Q84-induced caspase-3 cleavage. Co-overexpression of Exoc7^CC only^, but not NES-Exoc7^CC only^, suppressed the Prpf19’s modulatory effects. *n* = 3 biological replicas. Each *n* represents an independent preparation of cell protein samples. **c** Quantification of ATXN3-polyQ protein and cleaved caspase-3 protein levels in panel **b**. Error bars represent S.E.M. Statistical analysis was performed using one-way ANOVA followed by post hoc Tukey’s test. ns denotes no significant difference, * denotes *P* < 0.05 and ** denotes *P* < 0.01. Beta-tubulin was used as a loading control. Only representative images and blots are shown.

Exoc7 is a modular protein that is composed of a coiled-coil (CC) domain, an N-terminal domain, a middle domain, and a C-terminal domain (see ref. ^44^; Fig. 5a). Its CC domain has been implicated in interacting with Prpf19 (ref. ^44^). We generated an Exoc7 mutant construct that lacks the CC domain (Exoc7^∆CC^) to investigate the role of the CC domain of Exoc7 in the inhibition of the Prpf19 function (Fig. 5a). Unlike Exoc7^full-length^, we showed that Exoc7^∆CC^ co-expression was incapable of counteracting Prpf19 function in terms of downregulating ATXN3-Q84 protein level and suppressing caspase-3 cleavage (Fig. 5b, c). We next generated a construct that only expresses the Exoc7 CC domain, Exoc7^CC only^ (Fig. 5a), to further validate the role of the CC domain in regulating the Prpf19 function. Similar to Exoc7^full-length^, the co-expression of Exoc7^CC only^ was capable of abolishing the effect of Prpf19 on ATXN3-Q84 protein level and toxicity (Fig. 5b, c). We found that the localization of ATXN3-polyQ proteins did not change upon overexpression of Exoc7^full-length^ (Supplementary Fig. 5). Meanwhile, overexpressed Exoc7^full-length^ (Supplementary Fig. 5) and Exoc7^CC only^ (Fig. 6a) protein both localized to the cytosolic and nuclear compartments. Given that Prpf19 exerts its suppression effect on ATXN3-polyQ toxicity in the nuclear compartment (Fig. 3d–h), we further generated an NES-containing Exoc7^CC only^ construct (NES-Exoc7^CC only^; Fig. 5a) to investigate if the nuclear localization is critical for Exoc7^CC only^ to exert its inhibitory effect on Prpf19’s action on ATXN3-polyQ toxicity (Fig. 5b, c). By means of immunofluorescence, we confirmed that the NES-Exoc7^CC only^ protein fragment resided in the cytoplasm (Fig. 6a). Interestingly, when its localization was excluded from the cell nucleus, the NES-Exoc7^CC only^ fragment completely lost its inhibitory effect on Prpf19, the ATXN3-Q84 protein level was not downregulated and caspase-3 cleavage was not suppressed (Fig. 6b, c). This strongly suggests that the nuclear compartment allows Exoc7 to exert its modulatory effect on Prpf19 function. This is also in line with our results demonstrating that Prpf19 elicits its ATXN3-polyQ modifying effects in the nuclear subcellular compartment (Fig. 3d–h).

### Prpf19 and Exoc7 modulate SCA3 neurodegeneration in vivo

**Fig. 7:**
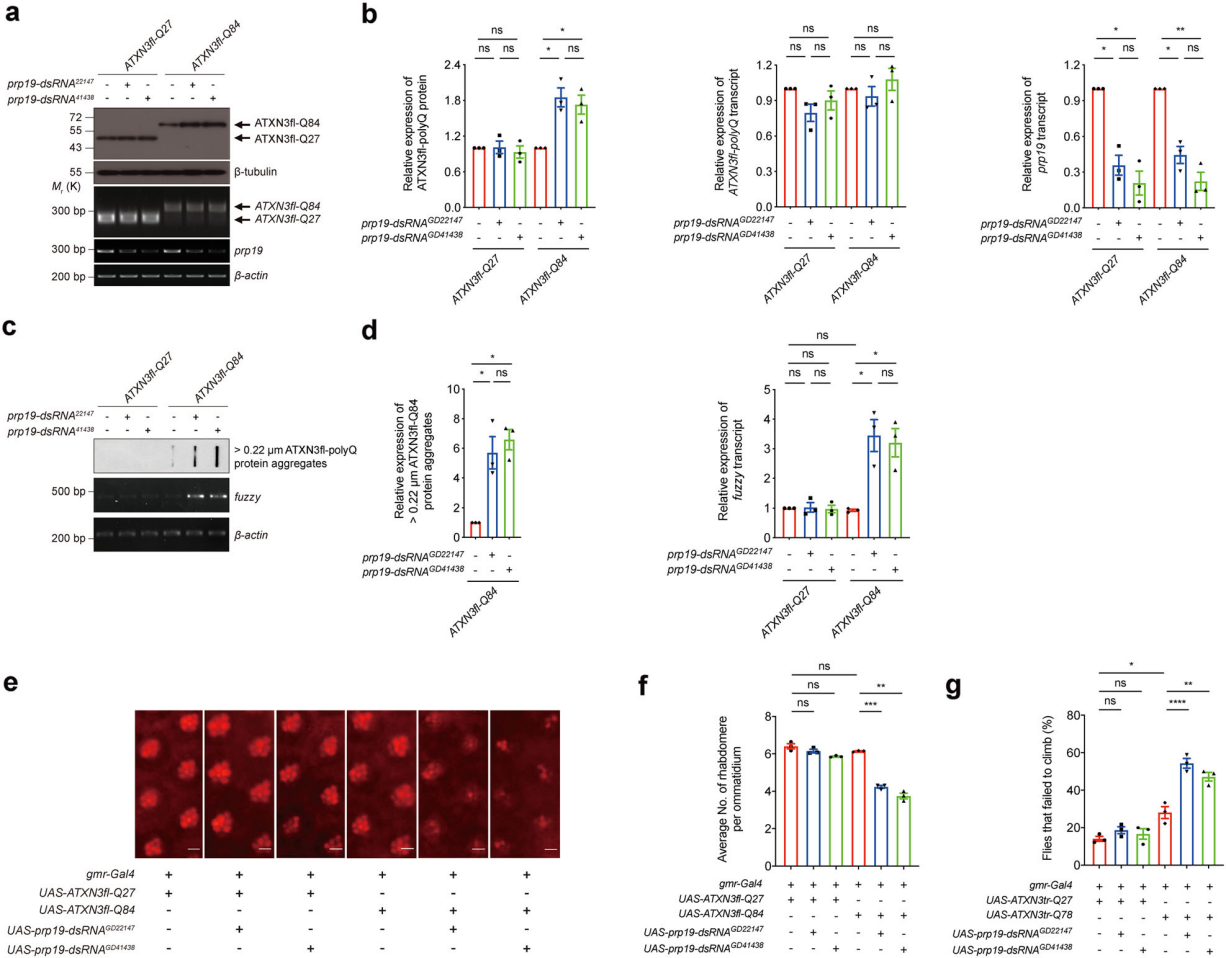
Knockdown of *prp19* enhances the expanded ATXN3-polyQ protein level and neurodegeneration in in vivo *Drosophila* model. **a** Knockdown of *prp19* enhanced the ATXN3fl-Q84 protein level, but it did not alter the levels of ATXN3fl-Q27 protein and *ATXN3-polyQ* transcripts. Two independent *prp19 double-strand RNA* (*dsRNA*) fly lines (GD22147 and GD41438) were used. The knockdown effect was confirmed by RT-PCR. *n* = 3 biological replicas. Each *n* represents an independent preparation of fly protein or RNA samples, in which ten fly heads were homogenized to extract proteins or RNAs in each control or experimental group. All flies were raised at 21.5 °C and assayed at 1-day post eclosion (dpe). **b** Quantification of the ATXN3-polyQ protein, *ATXN3-polyQ* transcript, and *prp19* transcript levels in panel **a**. Error bars represent S.E.M. Statistical analysis was performed using one-way ANOVA followed by post hoc Tukey’s test. ns denotes no significant difference, * denotes *P* < 0.05 and ** denotes *P* < 0.01. **c** Knockdown of *prp19* resulted in the increased levels of > 0.22 µm ATXN3fl-Q84 protein aggregates and *fuzzy* transcript. *n* = 3 biological replicas. Each *n* represents an independent preparation of fly protein or RNA samples, in which ten fly heads were homogenized to extract proteins or RNAs in each control or experimental group. All flies were raised at 21.5 °C and assayed at 1 dpe. **d** Quantification of the > 0.22 µm ATXN3fl-Q84 protein aggregates and fuzzy transcript levels in panel **c**. Error bars represent S.E.M. Statistical analysis was performed using one-way ANOVA followed by post hoc Tukey’s test. ns denotes no significant difference, * denotes *P* < 0.05. **e** Knockdown of *prp19* led to an enhancement of ATXN3fl-Q84 neurodegeneration in *Drosophila*. Two independent *prp19 dsRNA* fly lines (GD22147 and GD41438) were used. Scale bars: 5 μm. *n* = 3 biological replicas. Each *n* represents an indepe*n*dent trial of pseudo pupil assay, in which a total number of 20 fly eyes were examined, and 10 ommatidia per fly-eye were counted in each control or experimental group. All flies were raised at 21.5 °C and assayed at 1 dpe. **f** Quantification of the average number of rhabdomeres per ommatidium in panel **e**. Error bars represent S.E.M. Statistical analysis was performed using one-way ANOVA followed by post hoc Tukey’s test. ns denotes no significant difference, ** denotes *p* < 0.01 and *** denotes *p* < 0.001. The flies were of genotypes *w; gmr-Gal4 UAS-ATXN3fl-Q27*/ + *;* +/+, *w; gmr-Gal4 UAS-ATXN3fl-Q27*/*UAS-prp19-dsRNA*^*GD22147*^*;* +/+, *w; gmr-Gal4 UAS-ATXN3fl-Q27/*+*; UAS-prp19-dsRNA*^*GD41438*^/+, *w; gmr-Gal4*/+*; UAS-ATXN3fl-Q84*/ + , *w; gmr-Gal4*/*UAS-prp19-dsRNA*^*GD22147*^; *UAS-ATXN3fl-Q84*/ + , *w; gmr-Gal4*/+*; UAS-ATXN3fl-Q84*/*UAS-prp19-dsRNA*^*GD41438*^. **g** Knockdown of *prp19* enhanced the climbing defects in ATXN3tr-Q78 flies. *n* = 3 biological replicas. Each *n* represents an independent trial of the climbing assay, in which the climbing activity of at least 24 flies were measured in each biological replicate. All flies were raised at 25 °C and assayed at 5 dpe. Error bars represent S.E.M. Statistical analysis was performed using one-way ANOVA followed by post hoc Tukey’s test. ns denotes no significant difference, * denotes *P* < 0.05, ** denotes *P* < 0.01, and **** denotes *P* < 0.0001. The flies used for climbing assay were of genotypes *elav-Gal4/+; +/+; UAS-ATXN3tr-Q27/* + , *elav-Gal4/+; UAS-prp19-dsRNA*^*GD22147*^*/+; UAS-ATXN3tr-Q27/* + , *elav-Gal4/+; +/+; UAS-ATXN3tr-Q27/UAS-prp19-dsRNA*^*GD41438*^, *elav-Gal4/+; UAS-ATXN3tr-Q78/* + *;* + */* + , *elav-Gal4/+; UAS-ATXN3tr-Q78/UAS-prp19-dsRNA*^*GD22147*^*; +/+*, *elav-Gal4/+; UAS-ATXN3tr-Q78/* + *; UAS-prp19-dsRNA*^*GD41438*^*/+*. Beta-actin or beta-tubulin was used as a loading control. Only representative images, gels, and blots are shown.

**Fig. 8:**
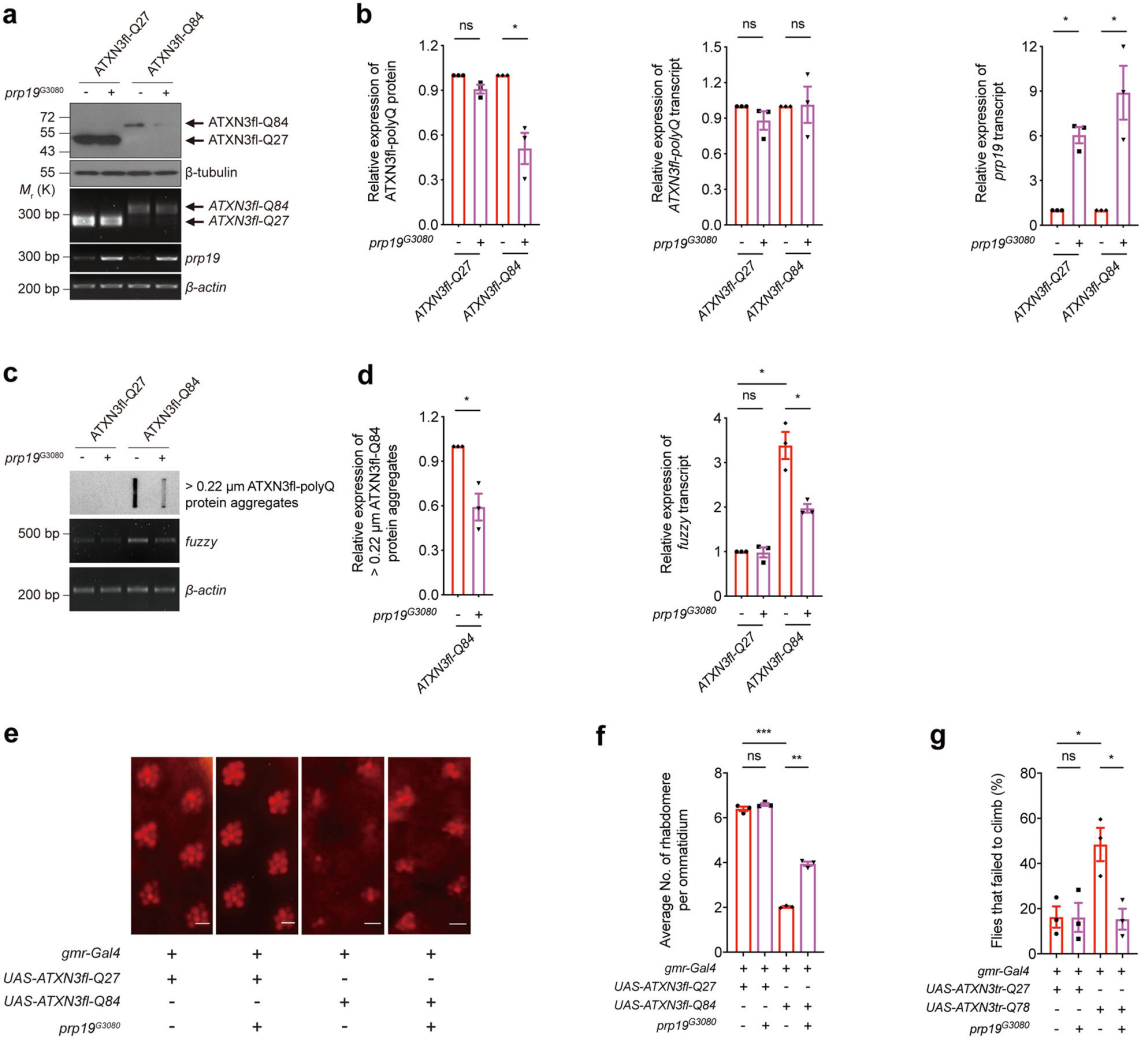
Overexpression of *prp19* suppresses the expanded ATXN3-polyQ protein level and neurodegeneration in in vivo *Drosophila* model. **a** Overexpression of *prp19* (*prp19*^*G3080*^) reduced the ATXN3fl-Q84 protein level, but it did not alter the levels of ATXN3fl-Q27 protein and *ATXN3-polyQ* transcripts. Overexpression of *prp19* was confirmed by RT-PCR. *n* = 3 biological replicas. Each *n* represents an independent preparation of fly protein or RNA samples, in which ten fly heads were homogenized to extract proteins or RNAs in each control or experimental group. All flies were raised at 21.5 °C and assayed at 12 dpe. **b** Quantification of the ATXN3-polyQ protein, *ATXN3-polyQ* transcript, and *prp19* transcript levels in panel **a**. Error bars represent S.E.M. Statistical analysis was performed using two-tailed unpaired Student’s *t* test. ns denotes no significant difference and * denotes *P* < 0.05. **c** Overexpression of *prp19* resulted in the decreased levels of > 0.22 µm ATXN3fl-Q84 protein aggregates and *fuzzy* transcript. *n* = 3 biological replicas. Each *n* represents an indepe*n*dent preparation of fly protein or RNA samples, in which ten fly heads were homogenized to extract proteins or RNAs in each control or experimental group. All flies were raised at 21.5 °C and assayed at 12 dpe. **d** Quantification of the > 0.22 µm ATXN3fl-Q84 protein aggregates and *fuzzy* transcript levels in panel **c**. Error bars represent S.E.M. Statistical analysis was performed using two-tailed unpaired Student’s *t* test. * denotes *P* < 0.05. **e** The ATXN3fl-Q84-induced neurodegeneration was suppressed by *prp19* overexpression (*prp19*^*G3080*^) in *Drosophila*. Scale bars: 5 μm. *n* = 3 biological replicas. Each *n* represents an independent trial of pseudo pupil assay, in which a total number of 20 fly eyes were examined, and 10 ommatidia per fly-eye were counted in each control or experimental group. All flies were raised at 21.5 °C and assayed at 12 dpe. **f** Quantification of the average number of rhabdomeres per ommatidium in panel **e**. Error bars represent S.E.M. Statistical analysis was performed using one-way ANOVA followed by post hoc Tukey’s test. ns denotes no significant difference, ** denotes *P* < 0.01 and *** denotes *P* < 0.001. The flies were of genotypes *w; gmr-Gal4 UAS-ATXN3fl-Q27*/ + *;* + / + , *w; gmr-Gal4 UAS-ATXN3fl-Q27*/*prp19*^*G3080*^*;* +/+, *w; gmr-Gal4*/+*; UAS-ATXN3fl-Q84*/ + , *w; gmr-Gal4*/*prp19*^*G3080*^; *UAS-ATXN3fl-Q84/* + . **g** Overexpression of *prp19* alleviated the climbing defects in ATXN3tr-Q78 flies. *n* = 3 biological replicas. Each *n* represents an independent trial of the climbing assay, in which the climbing activity of at least 20 flies was measured in each biological replicate. All flies were raised at 25 °C and assayed at 10 dpe. Error bars represent S.E.M. Statistical analysis was performed using one-way ANOVA followed by post hoc Tukey’s test. ns denotes no significant difference and * denotes *P* < 0.05. The flies used for climbing assay were of genotypes *elav-Gal4/+; +/+; UAS-ATXN3tr-Q27/* + , *elav-Gal4/+; prp19*^*G3080*^*/+; UAS-ATXN3tr-Q27/* + , *elav-Gal4/+; UAS-ATXN3tr-Q78/* + *;* + */* + , *elav-Gal4/+; UAS-ATXN3tr-Q78/prp19*^*G3080*^*; +/+*. Beta-actin or beta-tubulin was used as loading control. Only representative images, gels, and blots are shown.

We next made use of a transgenic *Drosophila* model of SCA3 to investigate the modulatory effect of Prpf19 in vivo. When the *Drosophila* ortholog of Prpf19, *pre-RNA processing factor 19* (*prp19*), was knocked down, both the soluble and aggregated levels of SCA3 disease protein, ATXN3fl-Q84, were increased (Fig. 7a–d). The accumulation of aggregated ATXN3-polyQ protein was found associated with transcriptional induction of a pro-apoptotic gene *fuzzy*^35^. Consistent with the increase of aggregated ATXN3fl-Q84 protein level, an upregulation of *fuzzy* transcript was detected upon *prp19* knockdown (Fig. 7c, d). Moreover, knockdown of *prp19* expression intensified retinal neurodegeneration, climbing defects, and reduced survival probabilities in SCA3 transgenic fly models (Fig. 7e–g, Supplementary Fig. 6 and Table 1). In contrast, overexpression of prp19 elicited the opposite ATXN3-polyQ toxicity-rescuing effect. The soluble and aggregated ATXN3fl-Q84 protein levels, along with the induction of *fuzzy*, were reduced in prp19-overexpressing flies (Fig. 8a–d). The retinal neurodegeneration, climbing defects, and compromised survival probabilities of SCA3 flies were coherently suppressed when prp19 was overexpressed (Fig. 8e–g, Supplementary Fig. 6, and Table 1). We further investigated the opposing modulatory effects of Exoc7 on expanded ATXN3-polyQ protein toxicity in vivo (Fig. 4). When the Exoc7 ortholog, exocyst 70 (*exo70*), was knocked down in *Drosophila*^37^, the soluble and aggregated ATXN3fl-Q84 protein levels were reduced (Supplementary Fig. 6a–d), similar to what we observed when *prp19* was overexpressed (Fig. 8a–d). Moreover, *exo70* knockdown also rescued ATXN3fl-Q84 neurodegeneration (Supplementary Fig. 6e, f).

**Table 1:** Summary of the fly survival assay results.

Days	ATXN3tr-Q27	ATXN3tr-Q27 x prp19-dsRNA^GD22147^	ATXN3tr-Q27 x prp19-dsRNA^GD41438^	ATXN3tr-Q27 x prp19^G3080^	ATXN3tr-Q78	ATXN3tr-Q78 x prp19-dsRNA^GD22147^	ATXN3tr-Q78 x prp19-dsRNA^GD41438^	ATXN3tr-Q78 x prp19^G3080^
3 dpe	100 + 0	100 + 0	100 + 0	100 + 0	100 + 0	100 + 0	100 + 0	100 + 0
6 dpe	100 + 0	100 + 0	96.296 + 1.625	100 + 0	93.077 + 2.226	93.750 + 2.140	96.923 + 1.515	97.561 + 1.391
9 dpe	100 + 0	100 + 0	95.556 + 1.774	100 + 0	88.462 + 2.802	89.063 + 2.759	90.000 + 2.631	97.561 + 1.391
12 dpe	97.727 + 1.297	99.200 + 0.797	93.333 + 2.147	97.414 + 1.474	83.846 + 3.228	80.469 + 3.504	82.308 + 3.347	95.935 + 1.781
15 dpe	94.697 + 1.950	94.400 + 2.056	91.852 + 2.355	92.241 + 2.484	80.000 + 3.508	69.531 + 4.068	71.538 + 3.958	90.244 + 2.675
18 dpe	93.182 + 2.194	90.400 + 2.635	90.370 + 2.539	88.793 + 2.929	73.077 + 3.890	64.063 + 4.241	64.615 + 4.194	86.992 + 3.033
21 dpe	91.667 + 2.406	90.400 + 2.635	88.889 + 2.705	88.793 + 2.929	72.308 + 3.925	62.500 + 4.279	61.538 + 4.267	82.927 + 3.393
24 dpe	89.394 + 2.680	84.800 + 3.211	86.667 + 2.926	83.621 + 3.436	67.692 + 4.102	57.031 + 4.376	55.385 + 4.360	80.488 + 3.573
27 dpe	87.879 + 2.841	81.600 + 3.466	85.926 + 2.993	83.621 + 3.436	64.615 + 4.194	50.781 + 4.419	50.000 + 4.385	75.610 + 3.872

## Discussion

In this study, we identified an E3 ligase, Prpf19, as a novel modulator of SCA3 toxicity, through which the ATXN3-polyQ disease protein was ubiquitinated, and ATXN3-polyQ-induced toxicity was suppressed (Fig. 1). Prpf19 was found to specifically interact with expanded ATXN3-polyQ protein via its C-terminal WD40 substrate recognition domain, an interaction that accounts for Prpf19’s modulatory effect on ATXN3-polyQ toxicity (Fig. 2). Interestingly, nuclear, but not cytoplasmic, Prpf19 is essential for modulating mutant ATXN3-polyQ protein levels and suppressing ATXN3-polyQ-induced toxicity (Fig. 3d–h). We further showed that Exoc7, a previously reported interacting partner of Prpf19 (ref. ^44^), perturbs the Prpf19’s E3 ligase activity and functions in the opposite way in modifying ATXN3-polyQ toxicity (Fig. 4). Both the nuclear localization and its CC domain is essential for Exoc7 to achieve the perturbation on Prpf19’s E3 ligase function (Figs. 5 and 6). Moreover, the modulatory effect of Prpf19 and Exoc7 was further confirmed in SCA3 *Drosophila* disease models in vivo (Figs. 7, 8 and Supplementary Fig. 6).

In this study, we focused on the E3 ligase function of Prpf19. We found that Prpf19 overexpression specifically promotes the ubiquitination of expanded ATXN3-polyQ protein, the reduction of expanded ATXN3-polyQ protein levels, and alleviation of ATXN3-polyQ-induced cytotoxicity were further observed (Fig. 1). A series of E3 ligases have been implicated in SCA3 pathogenesis via targeting distinct ATXN3-polyQ protein species for ubiquitination followed by degradation^12,18,47,48^. For instance, we recently reported that the F-box only protein 33 (FBXO33) enhances the ubiquitination of > 0.22 μm ATXN3-polyQ protein aggregates^18^. Unlike FBXO33, when Prpf19 was overexpressed, the ubiquitination level of soluble expanded ATXN3-polyQ protein was found to be increased (Fig. 1e, f). Interestingly, another U-box domain-containing E3 ligase, C-terminus of Hsp70-interacting protein (CHIP), was also found to modulate SCA3 pathology via targeting expanded ATXN3-polyQ protein for ubiquitination and degradation^49,50^. Similar to Prpf19, CHIP promotes the ubiquitination of soluble mutant ATXN3-polyQ protein^49^. In addition to F-box and U-box E3 ligases, several HECT domain-containing E3 ligases are also involved in SCA3 disease pathogenesis, and these HECT E3 ligases were shown to preferentially target soluble expanded ATXN3-polyQ protein for ubiquitination^10,51^. Therefore, these findings indicate that, in contrast to F-box E3 ligase, which ubiquitinates aggregated ATXN3-polyQ protein, U-box, and HECT E3 ligases exert their modulatory effects on soluble ATXN3-polyQ disease protein to achieve the modification of ATXN3-polyQ cytotoxicity. When expressed in neurons, expanded ATXN3-polyQ protein forms conformations with different levels of solubility^40,45^, our study, therefore, implies that neurons utilize different E3 ligases to degrade distinctive ATXN3-polyQ protein species in an attempt to combat SCA3 neurodegeneration.

Previous structural studies demonstrated that Prpf19 protein forms homo-oligomers to elicit its biological functions^22,52^. Vander Kooi et al. proposed a quaternary structural model that consists of a central tetrameric coiled-coil domain, flanked by the U-box domain dimers and four flexibly attached WD40 domains^22^. This model has been proven to be necessary for Prpf19 to mediate spliceosome assembly^52^, as well as exert its E3 ligase activity^22^. Interestingly, it was further demonstrated that amino acids 56–74 within Prpf19 protein are required for its self-interaction, and a synthetic peptide derived from Prpf19^56–74^ protein sequence was found to interfere with Prpf19 self-interaction and inhibit its pre-mRNA splicing function^52^. However, whether this short 19-amino acid sequence is necessary for Prpf19’s E3 ligase function is currently unknown. One interesting direction for further investigation may be to examine the role of Prpf19 homo-oligomerization in ATXN3-polyQ protein ubiquitination, degradation, and suppression of cytotoxicity.

In addition to the identification of Prpf19 as a novel modifier of ATXN3-polyQ toxicity, our findings also uncovered a mechanism for how Prpf19’s E3 ligase activity is modulated. We found that Exoc7, one of the binding partners of Prpf19 (ref. ^44^), diminishes Prpf19-mediated ATXN3-polyQ ubiquitination and degradation when co-overexpressed in cells (Fig. 4). Exoc7 belongs to the exocyst complex, which is required for targeting vesicles to the plasma membrane to govern cell adhesion, migration, and invasion^53–55^. Although originally identified as a cytosolic protein, Exoc7 was also found to shuttle to the cell nucleus, where it binds directly to Prpf19 and interferes with the pre-mRNA splicing^44^. Dellago et al. further showed that the N-terminal CC domain in Exoc7 (Exoc7^CC only^) is indispensable for the interaction with Prpf19 (ref. ^44^). In this study, we showed that the expression of Exoc7^CC only^ was also responsible for inhibiting the Prpf19’s E3 ligase activity, as demonstrated by the finding that co-overexpression of Exoc7^CC only^, but not Exoc7^ΔCC^, abolished Prpf19-mediated degradation of expanded ATXN3-polyQ protein (Fig. 5b, c). More interestingly, we found that the nuclear, but not cytoplasmic Exoc7^CC only^ is essential for carrying out such modification (Fig. 6). This is consistent with our findings that Prpf19-mediated modulation of expanded ATXN3-polyQ protein is restricted to the cell nucleus, and once localized to the cytoplasm, Prpf19 fails to exert any modulatory effect (Fig. 3d–h). The localization of mutant ATXN3-polyQ protein in the cell nucleus hinders the nuclear export machinery, leading to progressive accumulation of toxic ATXN3-polyQ protein and consequent cellular dysfunctions, including dysregulation of gene transcription, which contributes to neurodegeneration^29,56^. The UPS pathway is one of the major mechanisms to prevent the nuclear accumulation of toxic proteins^57^. However, in SCA3, nuclear UPS activity in neurons is impaired which results in the inefficient cleansing of mutant proteins^58^. The modifying effect of nuclear Prpf19 on ATXN3-polyQ toxicity and its associated regulatory mechanisms further demonstrate the cell nucleus as a pathogenic hot spot in SCA3.

Our results revealed that when co-overexpressed, the Exoc7^CC only^, but not Exoc7^ΔCC^ counteracts the Prpf19’s modification of ATXN3-polyQ toxicity, indicating the CC domain in Exoc7 is essential for exerting its regulatory effect on Prpf19’s E3 ligase function (Fig. 5). Previous work has shown that Exoc7^CC only^ interacts with the 68–90 amino acid sequence of Prpf19 protein, a region that overlaps with the Prpf19’s homo-oligomerization domain^44^. Since the formation of homo-oligomers is important for Prpf19 to elicit its E3 ligase function^22^, it would be of high interest to explore whether Exoc7 exploits the CC domain to disrupt the formation of homo-oligomeric Prpf19 to impair its E3 ligase activity. This would help further uncover the role of Prpf19 homo-oligomers in exerting its E3 ligase function and provide a new angle to investigate how the protein structure of Prpf19 is regulated to cause alteration of its biological function.

In summary, this study unveils the involvement of an E3 ligase Prpf19 in SCA3 disease pathogenesis and further demonstrates the modulatory mechanisms of Prpf19’s E3 function in mediating ATXN3-polyQ protein ubiquitination and degradation. These findings broaden our knowledge of the mechanistic basis of SCA3, especially the role of Prpf19-associated cellular pathways. In the meantime, we found that Prpf19 exerted a similar modifying effect on the mutant huntingtin-polyQ protein (Supplementary Fig. 2), suggesting a generic modulatory function of Prpf19 towards expanded polyQ domain-containing proteins. A future direction to enhance the impact of studying Prpf19 on polyQ diseases would be to further investigate the effect of Prpf19 on vertebrate models of polyQ disease in vivo. The development of potential small molecule or peptidylic activators that directly target Prpf19 will also provide therapeutic potentials against polyQ disease pathogenesis.

## Supplementary information

Supplementary Table 1

Supplementary Table 2

Supplementary Figure 1

Supplementary Figure 2

Supplementary Figure 3

Supplementary Figure 4

Supplementary Figure 5

Supplementary Figure 6

Supplementary Figure 7
