# Supplementary material for: A fine balance between Prpf19 and Exoc7 in achieving degradation of aggregated protein and suppression of cell death in spinocerebellar ataxia type 3
Source: Cell Death Dis. 2021 Feb 2;12(2):136. doi: 10.1038/s41419-021-03444-x (PMC7862454; doi:10.1038/s41419-021-03444-x)
Supplement: Supplementary file 1 — Supplementary Table 1 [file 41419_2021_3444_MOESM1_ESM.docx]

**Supplementary Table 1.** List of the antibodies used in co-immunoprecipitation assays

| **Figures** | **Antibodies used in IP** | **Antibodies used in IB** |
| --- | --- | --- |
| Fig. 2b | myc beads (20 μL; A7470, Sigma-Aldrich) | anti-flag (1:500; F3165, Sigma-Aldrich)  anti-myc (1:2 000; 2276, Cell Signaling Technology) |
| Fig. 2c | anti-DYKDDDDK (1:150; 2368, Cell Signaling Technology) | anti-myc (1:2 000; 2276, Cell Signaling Technology)  anti-flag (1:500; F3165, Sigma-Aldrich) |
| Supplementary Fig. 3a | anti-myc (1:200; 2276, Cell Signaling Technology) | anti-Prpf19 (1:1 000; A300-102A, Bethyl Laboratories, Inc.)  anti-myc (1:500; 2278, Cell Signaling Technology) |
