# Supplementary material for: A fine balance between Prpf19 and Exoc7 in achieving degradation of aggregated protein and suppression of cell death in spinocerebellar ataxia type 3
Source: Cell Death Dis. 2021 Feb 2;12(2):136. doi: 10.1038/s41419-021-03444-x (PMC7862454; doi:10.1038/s41419-021-03444-x)
Supplement: Supplementary file 2 — Supplementary Table 2 [file 41419_2021_3444_MOESM2_ESM.docx]

**Supplementary Table 2.** List of the antibodies used in ubiquitination assays

| **Figures** | **Antibodies used in IP** | **Antibodies used in IB** |
| --- | --- | --- |
| Fig. 1e | anti-GFP (1:500; 632381, Clontech) | anti-HA (1:1 000; H6908, Sigma-Aldrich)  anti-GFP (1:1 000; ab6556, Abcam)  anti-flag (1:1 000; F3165, Sigma-Aldrich)  anti-GFP (1:2 000; 632381, Clontech) |
| Fig. 1g | anti-GFP (1:500; 632381, Clontech) | anti-HA (1:1 000; H6908, Sigma-Aldrich)  anti-GFP (1:1 000; ab6556, Abcam)  anti-Prpf19 (1:1 000; ab27692, Abcam)  anti-GFP (1:2 000; 632381, Clontech) |
| Fig. 4c | anti-GFP (1:500; 632381, Clontech) | anti-HA (1:1 000; H6908, Sigma-Aldrich)  anti-GFP (1:1 000; ab6556, Abcam)  anti-myc (1:2 000; 2276, Cell Signaling Technology)  anti-flag (1:1 000; F3165, Sigma-Aldrich)  anti-GFP (1:2 000; 632381, Clontech) |
| Supplementary Fig. 2c | anti-flag (1:400; F3165, Sigma-Aldrich) | anti-HA (1:1 000; H6908, Sigma-Aldrich)  anti-DYKDDDDK (1:1 000; 2368, Cell Signaling Technology)  anti-Prpf19 (1:1 000; ab27692, Abcam)  anti-flag (1:200; F3165, Sigma-Aldrich) |
| Supplementary Fig. 3f | anti-myc (1:200; 2276, Cell Signaling Technology) | anti-HA (1:1 000; H6908, Sigma-Aldrich)  anti-myc (1:500; 2278, Cell Signaling Technology)  anti-Prpf19 (1:1 000; ab27692, Abcam)  anti-myc (1:2 000; 2276, Cell Signaling Technology) |
