# Supplementary figures and images for: A fine balance between Prpf19 and Exoc7 in achieving degradation of aggregated protein and suppression of cell death in spinocerebellar ataxia type 3
Source: Cell Death Dis. 2021 Feb 2;12(2):136. doi: 10.1038/s41419-021-03444-x (PMC7862454; doi:10.1038/s41419-021-03444-x)

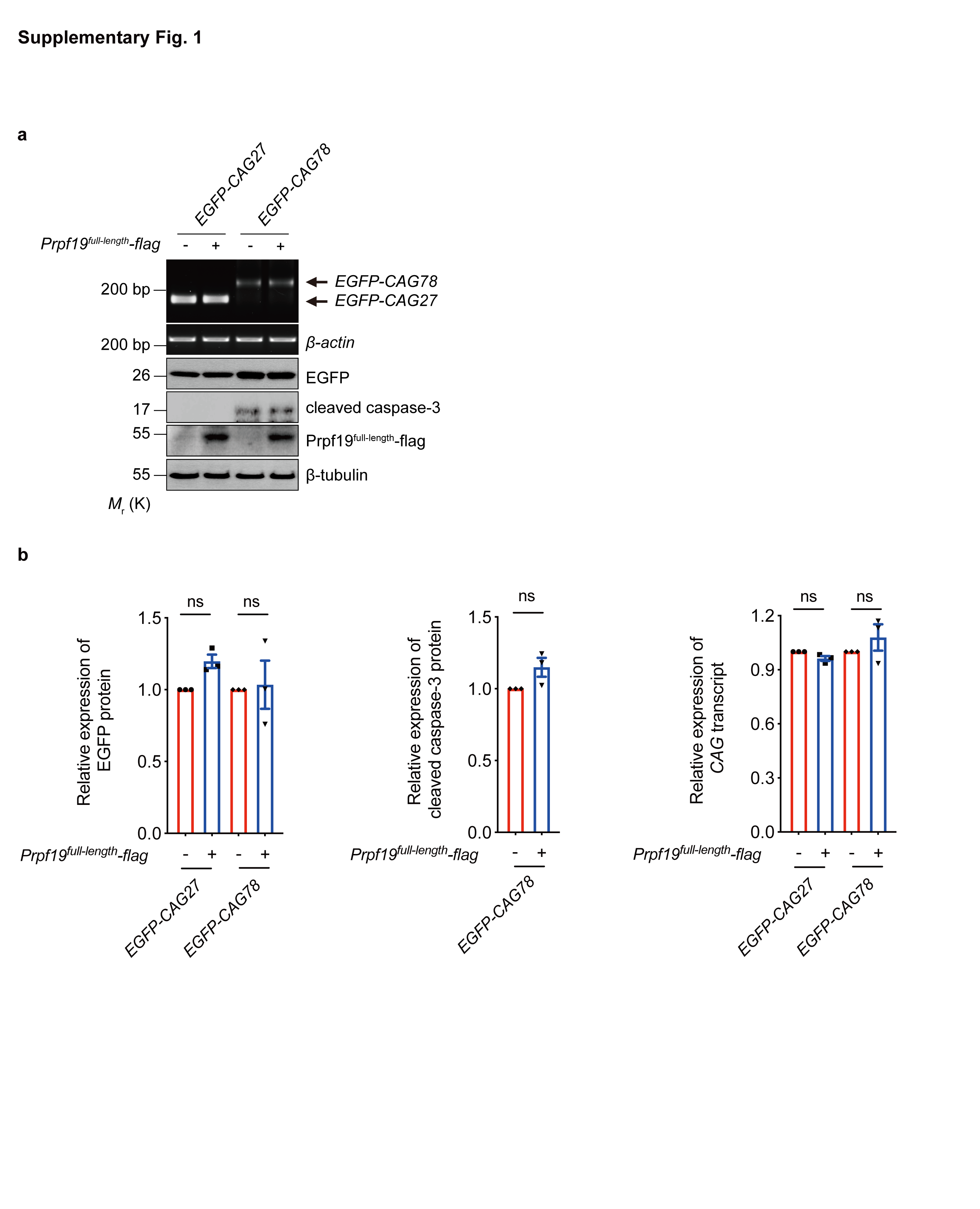

Supplement: Supplementary file 3 — Supplementary Figure 1 [file 41419_2021_3444_MOESM3_ESM.png]

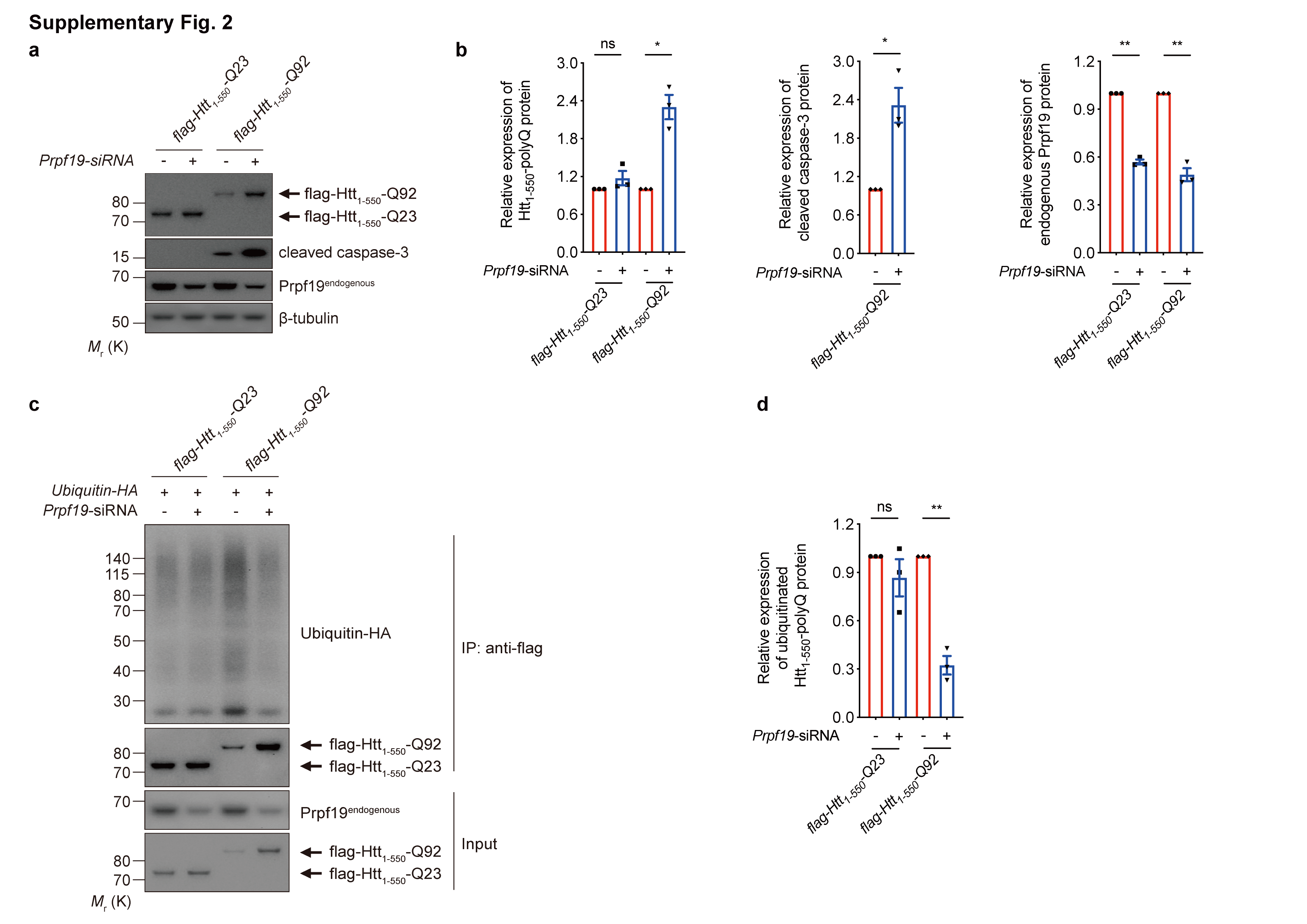

Supplement: Supplementary file 4 — Supplementary Figure 2 [file 41419_2021_3444_MOESM4_ESM.png]

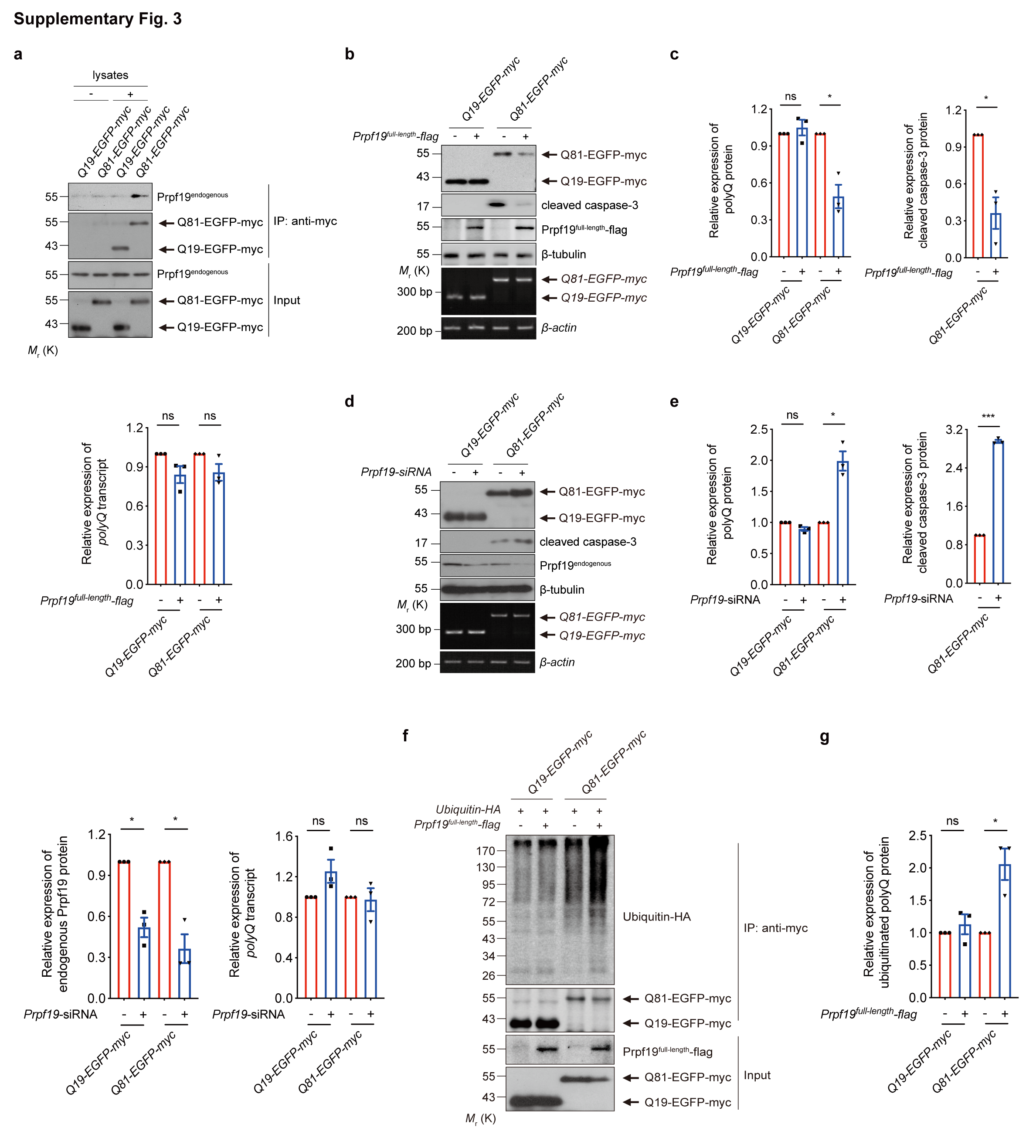

Supplement: Supplementary file 5 — Supplementary Figure 3 [file 41419_2021_3444_MOESM5_ESM.png]

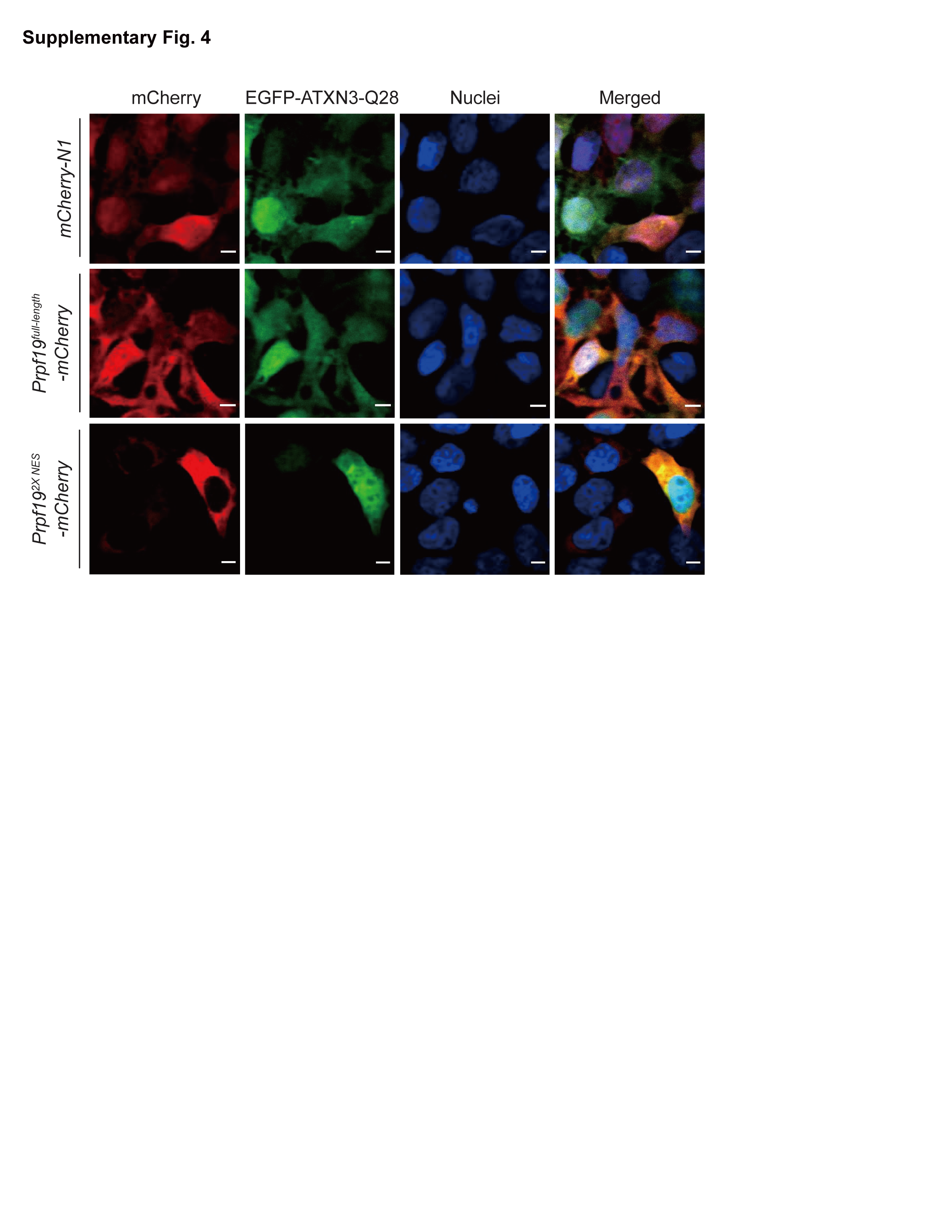

Supplement: Supplementary file 6 — Supplementary Figure 4 [file 41419_2021_3444_MOESM6_ESM.png]

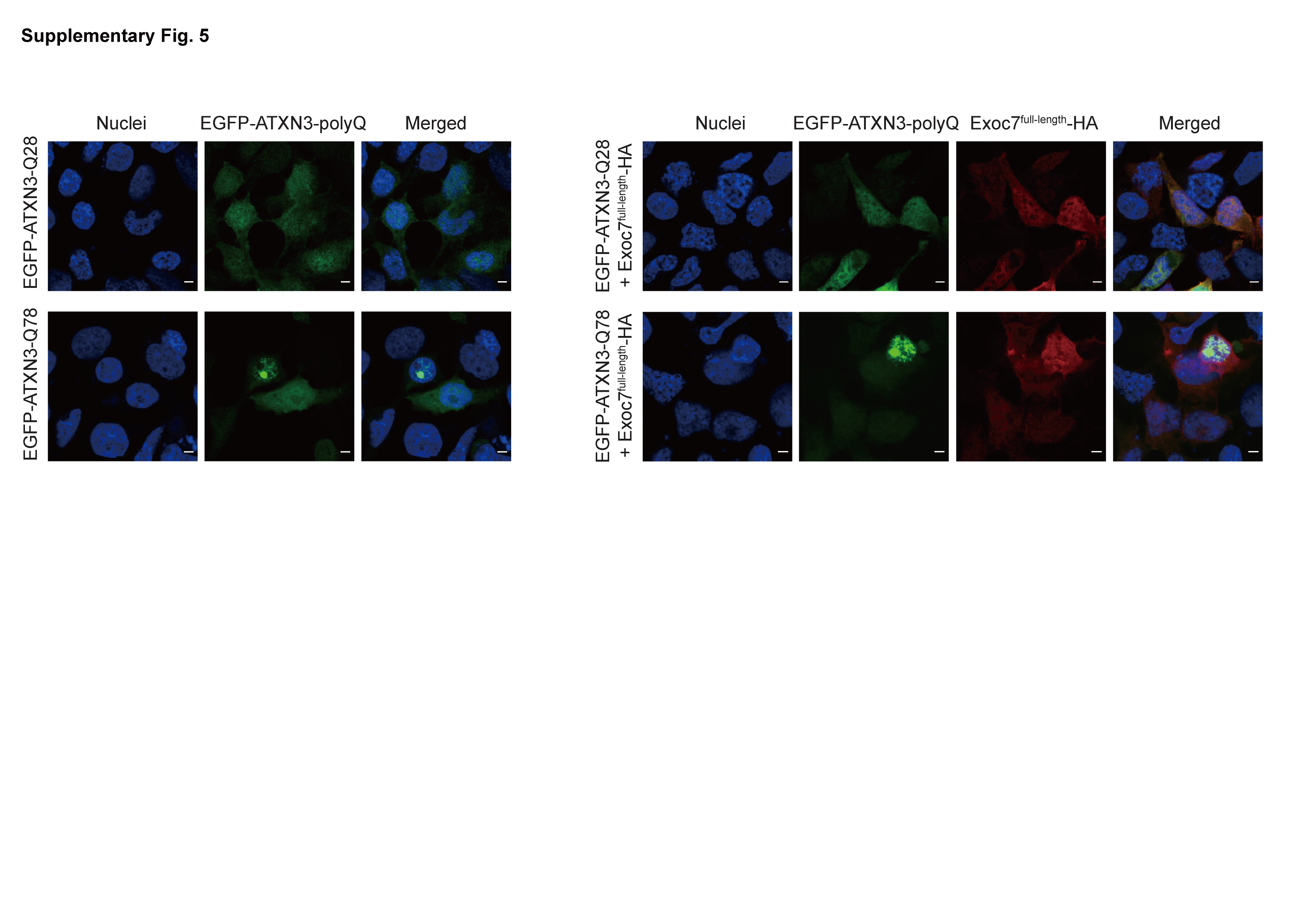

Supplement: Supplementary file 7 — Supplementary Figure 5 [file 41419_2021_3444_MOESM7_ESM.png]

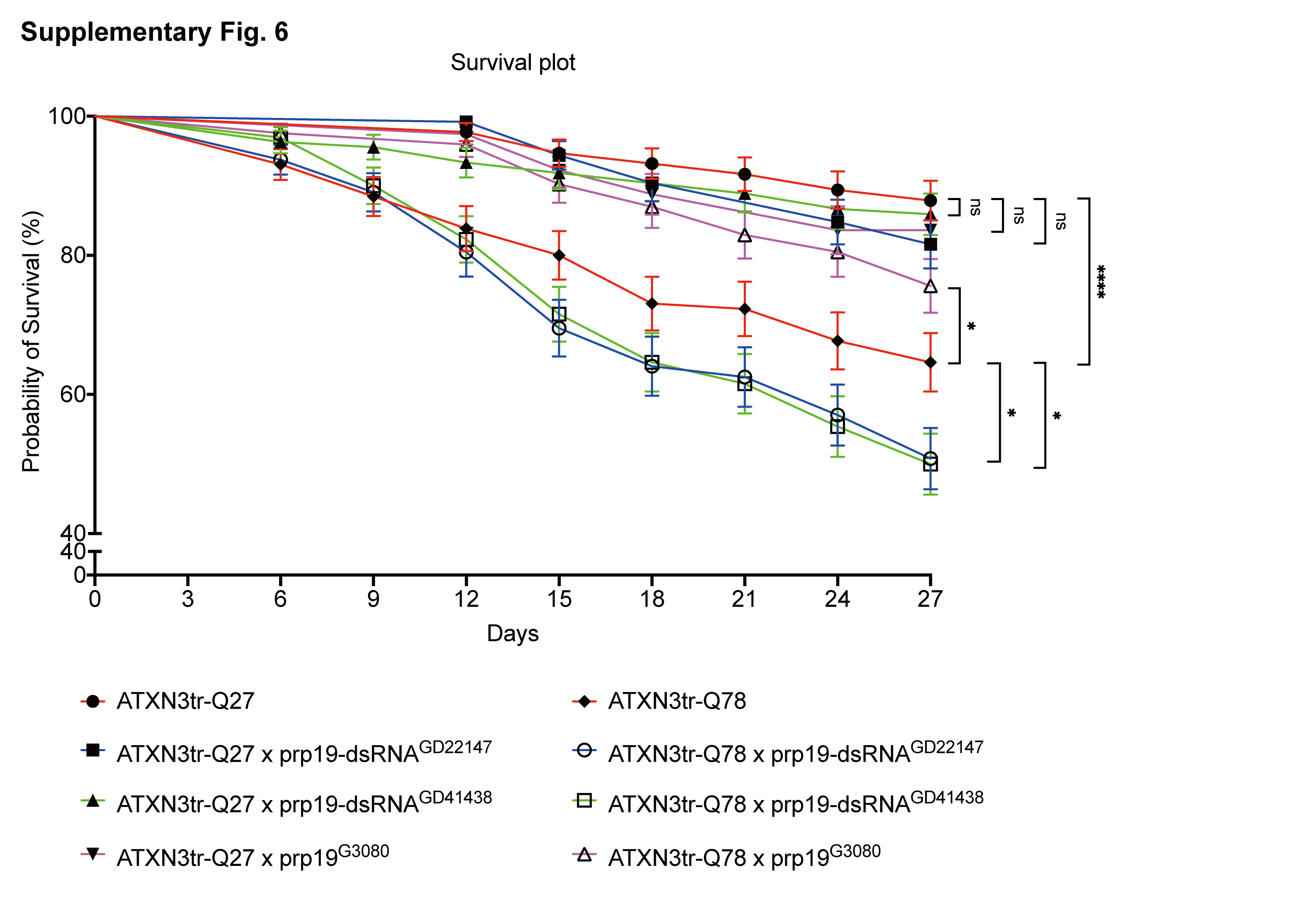

Supplement: Supplementary file 8 — Supplementary Figure 6 [file 41419_2021_3444_MOESM8_ESM.png]

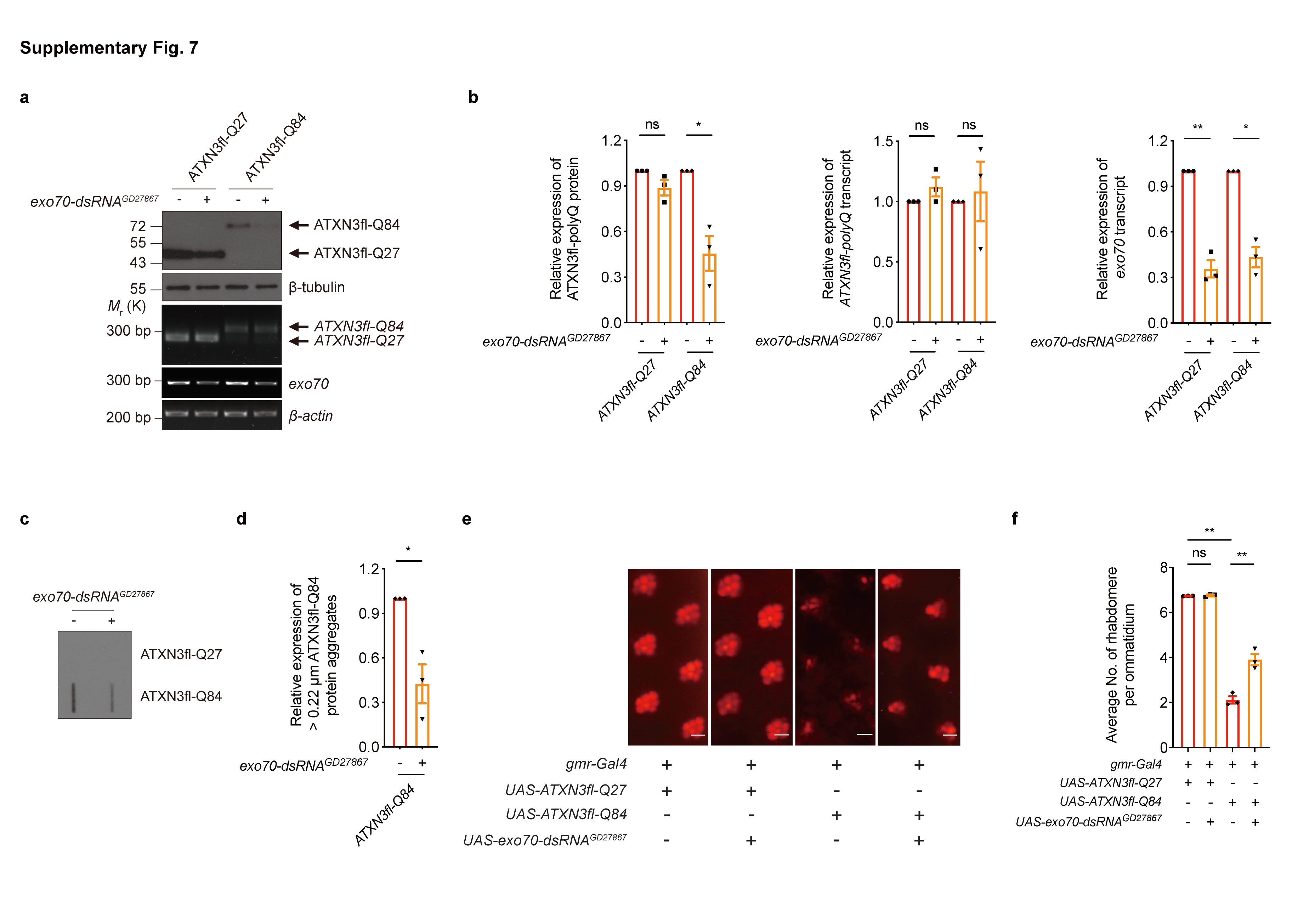

Supplement: Supplementary file 9 — Supplementary Figure 7 [file 41419_2021_3444_MOESM9_ESM.png]
